# Use of electron microscopy to determine presence of coal dust in a neighborhood bordering an open-air coal terminal in Curtis Bay, Baltimore, Maryland, USA

**DOI:** 10.1016/j.scitotenv.2024.176842

**Published:** 2024-10-11

**Authors:** Matthew A. Aubourg, Kenneth J.T. Livi, Gregory G. Sawtell, Carlos C. Sanchez-Gonzalez, Nicholas J. Spada, Russell R. Dickerson, Wen-An Chiou, Conchita Kamanzi, Gurumurthy Ramachandran, Ana M. Rule, Christopher D. Heaney

**Affiliations:** a Community Science and Innovation for Environmental Justice (CSI EJ) Initiative, Department of Environmental Health and Engineering, Johns Hopkins Bloomberg School of Public Health, Baltimore, MD 21205, United States; b Department of Environmental Health and Engineering, Johns Hopkins Bloomberg School of Public Health, Baltimore, MD 21205, United States; c Materials Characterization and Processing, Department of Materials Science and Engineering, Johns Hopkins University, Baltimore, MD 21211, United States; d Community of Curtis Bay Association, Baltimore, MD 21226, United States; e South Baltimore Community Land Trust, Baltimore, MD 21225, United States; f Air Quality Research Center, University of California, Davis, CA 95616, United States; g Department of Atmospheric and Oceanic Science, University of Maryland, College Park, MD 20742, United States; h Advanced Imaging and Microscopy Laboratory (AIM Lab), Maryland NanoCenter, University of Maryland, College Park, MD 20742, United States; i Department of Chemical Engineering, Minerals to Metals Initiative, University of Cape Town, Cape Town, 7701, South Africa; j Institute of Infectious Diseases and Molecular Medicine, University of Cape Town, 7925, South Africa; k Department of Epidemiology, Johns Hopkins Bloomberg School of Public Health, Baltimore, MD 21205, United States; l Department of International Health, Johns Hopkins Bloomberg School of Public Health, Baltimore, MD 21205, United States

**Keywords:** Coal dust, Coal terminal, Dust characterization, SEM-EDX, Community-driven research, Environmental justice

## Abstract

**Background:**

Despite decreasing US consumption, over 90 million metric tons of coal were exported by the US in 2023, requiring significant infrastructure for transport, handling, and storage of coal at export terminals. Residents in Curtis Bay, Baltimore, Maryland, USA live at the fenceline of an open-air coal terminal and have, for decades, reported rapid accumulation of black dust at their homes. Community-level exposure to coal dust originating from coal handling and storage terminals has remained largely unexplored.

**Objectives:**

To investigate community-identified concerns and use a community-driven approach to determine the presence/absence of coal dust on Curtis Bay surfaces.

**Methods:**

Passive settled dust samples were collected from two residential areas, 345 m and 1235 m from the coal terminal, using conductive carbon tape. Scanning electron microscopy and energy dispersive X-ray spectroscopy (SEM-EDX) of standard reference coal material and positive control material from the coal terminal in Curtis Bay were used to optimize the morphological and elemental classification criteria for coal dust. A manual SEM-EDX protocol was developed to identify coal particles in settled dust collected on conductive carbon tape in community settings.

**Results:**

SEM-EDX analysis confirmed presence of coal dust sampled at both residential locations. Estimated coal dust particle loading at the proximal and distal site were 13.2 and 3.4 coal particles/mm^2^, respectively. The coal dust particles identified met specific criteria, including size (>5 μm), morphology, and elemental composition (≥75 % carbon, ≤20 % oxygen).

**Discussion:**

These findings are consistent with longstanding community concerns and lived experiences regarding the presence of coal dust in Curtis Bay, which neighbors a major open-air coal terminal. This approach has potential for other communities neighboring coal terminals to assess similar concerns with residential coal dust exposure.

## Graphical Abstract

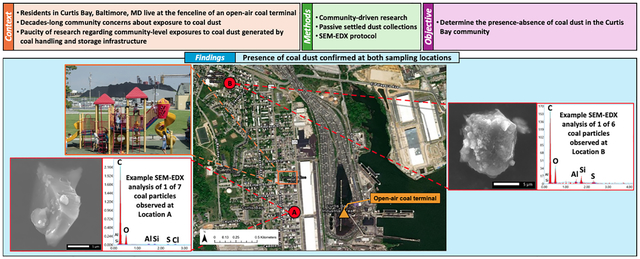

## 1. Introduction

Despite waning consumption of fossil fuels in the United States (US), the global consumption of coal reached a record high in 2023 (Energy Institute, 2023; International Energy Agency (IEA), 2026). In the US, significant amounts of coal–over 90 million metric tons in 2023–are destined for export (U.S. Energy Information Administration (EIA), 2024a). This material is stockpiled at coal terminals where there is substantial infrastructure in place for coal transport, handling, and storage.

Recent studies have identified coal transport as a significant contributor to local fine particulate matter air pollution (Ostro et al., 2023a; Ostro et al., 2023b; Jaffe et al., 2015) and increased mortality and morbidity (Ostro et al., 2024). Jha and Muller (2018) found that coal handling and storage at coal stockpiles increase adult and infant mortality by 1.1 % and 3.2 %, respectively, for every 10 % increase in fine particulate matter (particles of aerodynamic diameter ≤ 2.5 μm or PM_2.5_) generated within an approximately 40 km radius (Jha and Muller, 2018). Coal terminals, transport infrastructure, and other industrial operations are often sited in low-income communities and communities of color, contributing to both public health and environmental justice concerns. Most of the scientific literature on coal exposure has focused on occupational exposures of coal miners (Petsonk et al., 2013; Hendryx et al., 2020a; Liu and Liu, 2020; Leonard et al., 2020) with little research addressing harmful exposure to coal dust in communities near coal mining areas. Studies have determined that areas and communities proximal to coal mines have elevated exposure to particulate matter and heavy metals (Rout et al., 2013; Kurth et al., 2014; Hendryx et al., 2020b; Huertas et al., 2012), adversely impacting health outcomes and quality of life (Higginbotham et al., 2010; Cortes-Ramirez et al., 2018; Hendryx, 2009; Hendryx and Entwhistle, 2015). In communities proximal to open-air handling and storage of other materials (distal from extraction activities), the presence of fugitive dust has been documented both by fenceline residents’ testimonies and via exposure studies (Ostro et al., 2023b; Hendryx et al., 2016; Jereb et al., 2009; Sorte et al., 2018). Despite this, there remains a paucity of research regarding community-level exposure to coal dust and associated environmental health impacts originating from coal handling and storage infrastructure, not in proximity to coal mining areas.

**Fig. 1.**
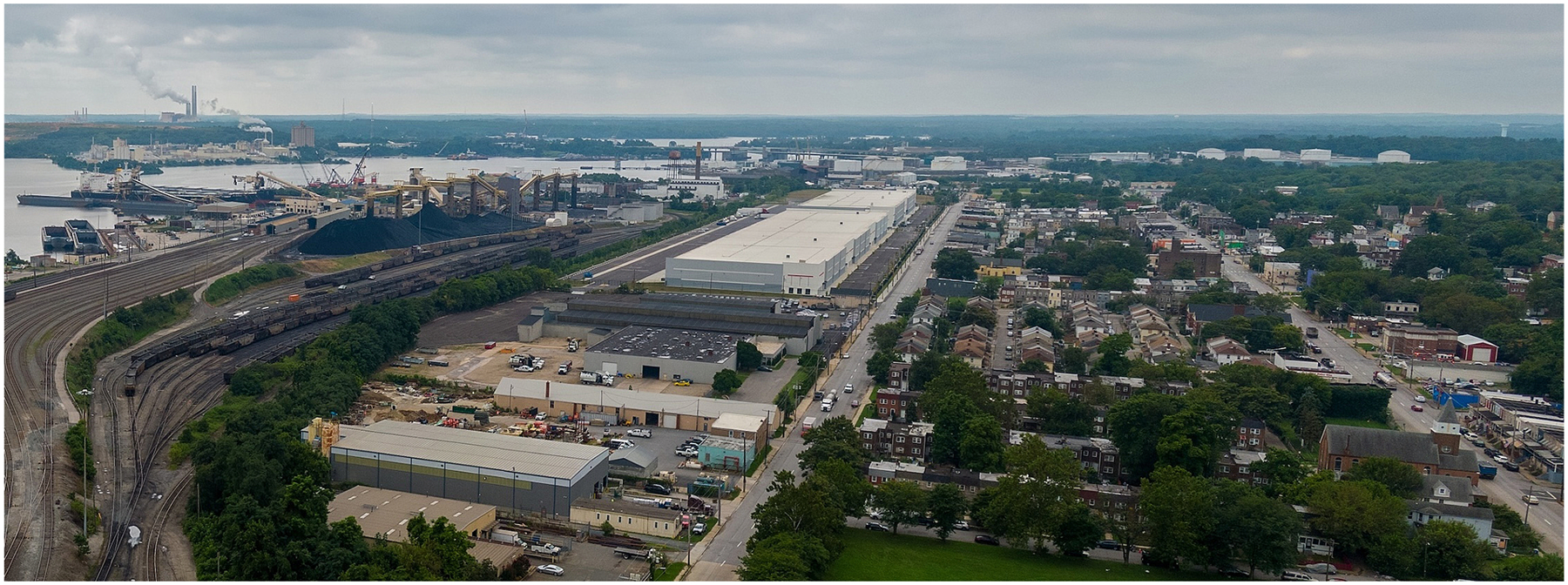
Drone photograph of the community of Curtis Bay, Baltimore, Maryland, USA, depicting industrial facilities and activities, including the open-air coal terminal and heavy diesel truck traffic, proximal to residential areas. Image credit to Ryan Gattis, October 26, 2023.

In 2023, Baltimore, Maryland was the second largest exporter of coal in the US, with approximately 30 million metric tons of coal leaving the Port of Baltimore, a nearly eight-fold increase since 2002 (U.S. Energy Information Administration (EIA), 2024b). The open-air coal terminal operated by CSX Corporation in Curtis Bay, South Baltimore, has an annual throughput capacity of over 12 million metric tons of coal “with room to grow” (CSX Corporation, n.d.). Alongside homes, local schools, and community spaces, a high density of industrial activity is located in Curtis Bay. These include the open-air coal terminal, heavy diesel truck traffic (Dickerson et al., n.d.), the largest medical waste incinerator in the US (Maryland Department of the Environment, 2024), oil and gas processing and storage, and other industrial land uses. The open-air coal terminal is located approximately 300 m from nearby homes and the community’s recreation center (Fig. 1).

Residents in Curtis Bay living at the fenceline of this coal terminal have raised concerns about numerous environmental health issues, including exposure to coal dust in their neighborhood. For decades, Curtis Bay residents have reported black dust on outdoor and indoor surfaces of their homes and property (Dickerson et al., n.d.; Community of Curtis Bay Association (CCBA) et al., 2023; South Baltimore Community Land Trust (SBCLT), 2023; Sierra Club Maryland Chapter, 2013). On December 30, 2021, a major explosion at the coal terminal blasted coal dust several blocks into the Curtis Bay community, shattered windows of nearby homes, and caused panic throughout the South Baltimore area (Kazanjian, 2022; South Baltimore Community Land Trust, 2023). This emergency event also energized action and coordination efforts to investigate the community health and quality of life impacts of living at the fenceline of the open-air coal terminal during its routine day-to-day operation (Community of Curtis Bay Association (CCBA) et al., 2023; Aubourg et al., 2023). Curtis Bay residents have recorded on camera fugitive dust emissions and activities at the coal terminal since the 2021 explosion (Shen, 2024; South Baltimore Community Land Trust (SBCLT), 2024a). In the following sections, we describe the development and application of a protocol to determine the presence-absence of coal dust in passive settled dust samples collected in the Curtis Bay community.

## 2. Materials and methods

### 2.1. Study setting

Following the December 2021 explosion at the coal terminal, the Community of Curtis Bay Association (Community of Curtis Bay Association (CCBA), 2024) and South Baltimore Community Land Trust (South Baltimore Community Land Trust (SBCLT), 2024b) initiated a collaboration with neighboring community-based organizations, the Maryland Department of the Environment (MDE) Air and Radiation Administration, researchers from the Department of Environmental Health and Engineering at the Johns Hopkins Bloomberg School of Public Health (BSPH), the Johns Hopkins University Materials Characterization and Processing Facility (JHU-MCP), and the Department of Atmospheric and Oceanic Sciences and the Advanced Imaging and Microscopy Laboratory at the University of Maryland (UMD). In December 2023, this collaborative team released a report of preliminary findings from a community-led investigation of the presence of coal dust in the Curtis Bay community, utilizing neighborhood-level air monitoring, statistical source apportionment techniques, and preliminary analysis (scanning electron microscopy and energy dispersive X-ray spectroscopy [SEM-EDX]) of passive settled dust samples (Community of Curtis Bay Association (CCBA) et al., 2023). SEM imaging allows for determination of the size and morphology of particles while EDX analysis provides particle elemental composition. Following the December 2023 report, additional exposure and coal characterization experts from the University of California Davis Air Quality Research Center and the University of Cape Town Minerals to Metals Initiative supported further development of analytical procedures described herein.

### 2.2. Sample collection

**Fig. 2.**
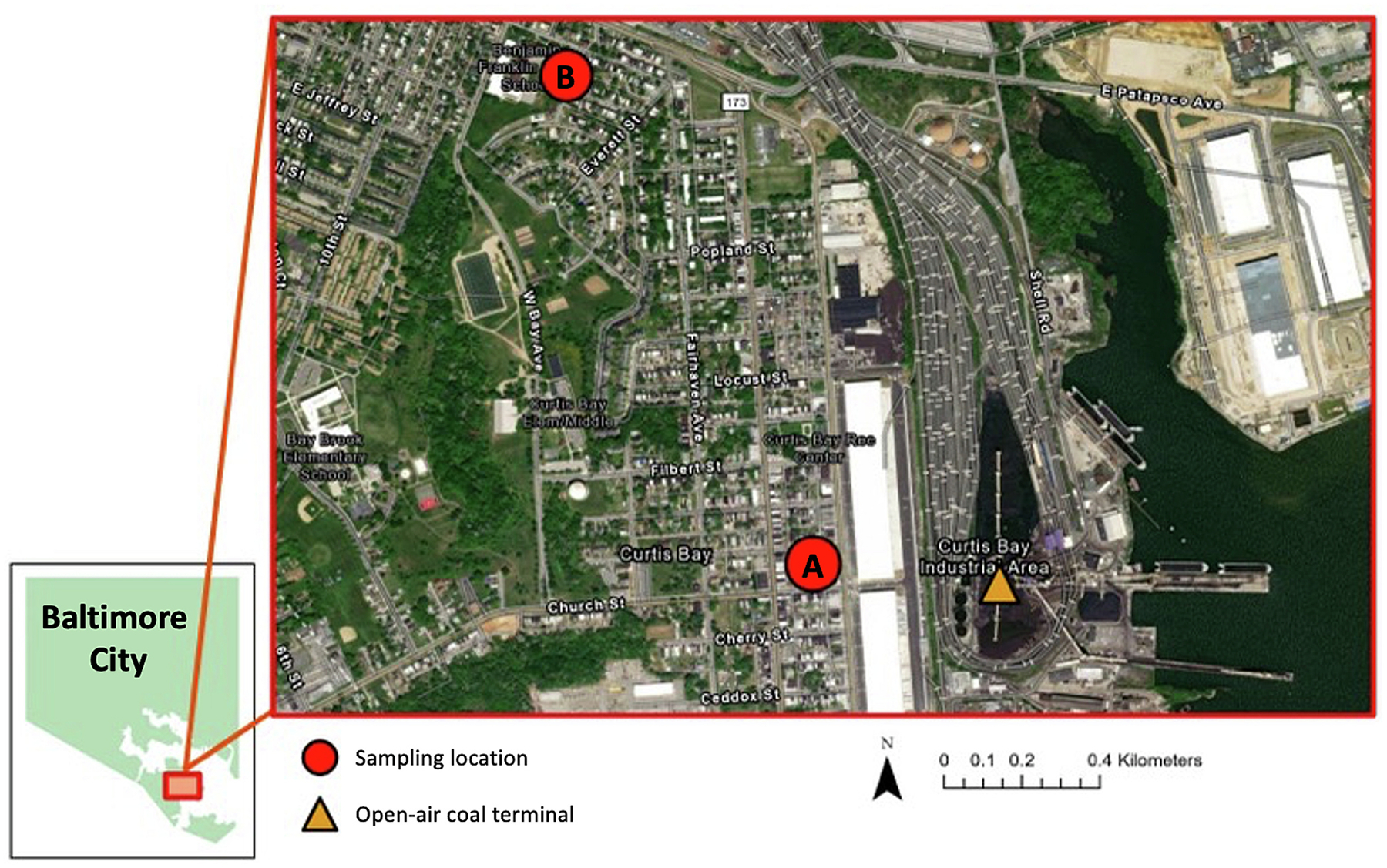
Approximate passive settled dust sampling locations in residential areas of the Curtis Bay community (red circles) and the open-air coal terminal (orange triangle). Basemap source: Esri Community Map Contributors, City of Baltimore, Baltimore County Government, County of Anne Arundel, VGIN, Esri, TomTom, Garmin, SafeGraph, GeoTechnologies, Inc., METI/NASA.

Sampling was conducted in residential areas of Curtis Bay in South Baltimore, Maryland, USA. Samples were collected 345 m (Location A) and 1235 m (Location B) from the centroid of the coal terminal property boundary (Fig. 2). Location A was near several homes, a community recreation center, and two major residential diesel truck routes. Location B, further from the coal terminal, was near the local elementary and high schools, within a dense residential area, and near community green spaces.

Field sampling kits included an 8 × 40 mm^2^ segment of double-sided, adhesive conductive carbon tape (Ted Pella, Inc., Redding, CA, US) sealed inside a labeled PetriSlide (Millipore, Merck KGaA, Darmstadt, DE). At each sampling location, a member of the study team adhered the segment of carbon tape to an outdoor, horizontal surface using gloves and tweezers cleaned with 70 % ethanol. Surfaces were cleaned before adhering the tape. The carbon tape was left exposed to collect settled dust for a three-day period (October 26–29, 2023) in which the coal terminal was in operation. The three-day sampling period was selected to reflect community members’ decades-long observations of rapid black dust accumulation at their homes. A laboratory blank was obtained during assembly of sampling kits by exposing carbon tape to ambient air in the biosafety cabinet where all sampling kits were assembled. A field blank was obtained at each sampling location by exposing the carbon tape to ambient air for 30 s. Following the three-day sampling period, a member of the study team carefully removed the conductive carbon tape from its surface and resealed it in the corresponding PetriSlide, using new gloves and tweezers cleaned with 70 % ethanol. PetriSlides were sealed with tape and placed in individual packaging prior to, during, and following the sampling period. Sealed samples were stored at room temperature until analysis.

### 2.3. SEM-EDX analytical protocol

#### 2.3.1. SEM-EDX instrumentation

Samples were analyzed in the JHU-MCP using a JEOL JSM-IT700HR InTouchScopeTM Field Emission SEM (JEOL US, Inc., Peabody, MA, US). Imaging was exclusively performed by secondary electrons. Energy dispersive X-ray analysis was conducted using an EDAX Octane Elect Plus silicon drift detector (Ametek, Inc., Berwyn, PA, US). Semi-quantitative analyses were performed using the EDAX APEX software using a supplied ZAF analysis routine.

Results for the optimization of coal particle classification criteria were independently confirmed via SEM (Hitachi SU-70 Schottky Field Emission Gun SEM; Hitachi High-Tech Corporation, Minato-ku, Tokyo, JPN) and EDX (Bruker XFlash 6160; Bruker Corporation, Billerica, MA, US) at UMD.

Elemental composition analyses via EDX presented here are semi-quantitative in accuracy and precision. No attempt was made to account for particle shape effects on differential absorption, shadow effects of large particle interferences, and thickness effects due to particle size. These effects will increase the variability of the analyses and possibly shift carbon‑oxygen ratios.

#### 2.3.2. Optimization of coal particle classification criteria

To evaluate the potential influence of the carbon tape substrate and optimize the elemental classification criteria for a coal particle, we conducted comparative SEM-EDX analyses of two positive control coal materials on a beryllium planchette (essentially free from carbon) and conductive carbon tape. This methodology was informed by standard practice in auto-SEM systems. Analyses via EDX were collected for 30 s per particle. Positive controls included National Institute of Standards and Technology (NIST) subbituminous coal standard reference material (SRM; 2682c) and a composite coal sample from the coal terminal in Curtis Bay. The SEM was operated under high vacuum conditions at 15 kV accelerating voltage and an ideal working distance of approximately 10 mm. Beam current ranged from 15 to 35 nA to optimize for SEM-EDX analysis of coal particles. Particle size by length of longest visible dimension (L) was obtained using ImageJ software (Schneider et al., 2012). Observed particles, ranging from approximately L = 500 nm to 100 μm, were analyzed for carbon, oxygen, sodium, magnesium, aluminum, silicon, phosphorus, sulfur, calcium, and iron. Three criteria were used to characterize coal particles: size, elemental composition, and morphology.

##### 2.3.2.1. Particle size and elemental composition.

Coal dust contains a heterogenous mixture of carbonaceous, mineral, and other particle types with varying levels of carbon and oxygen content (Raju and Rom, 1998). Elemental compositional analysis of carbon tape without coal had carbon content of 94 atomic weight percent (At.%) and oxygen content of 6 At.%.

**Fig. 3.**
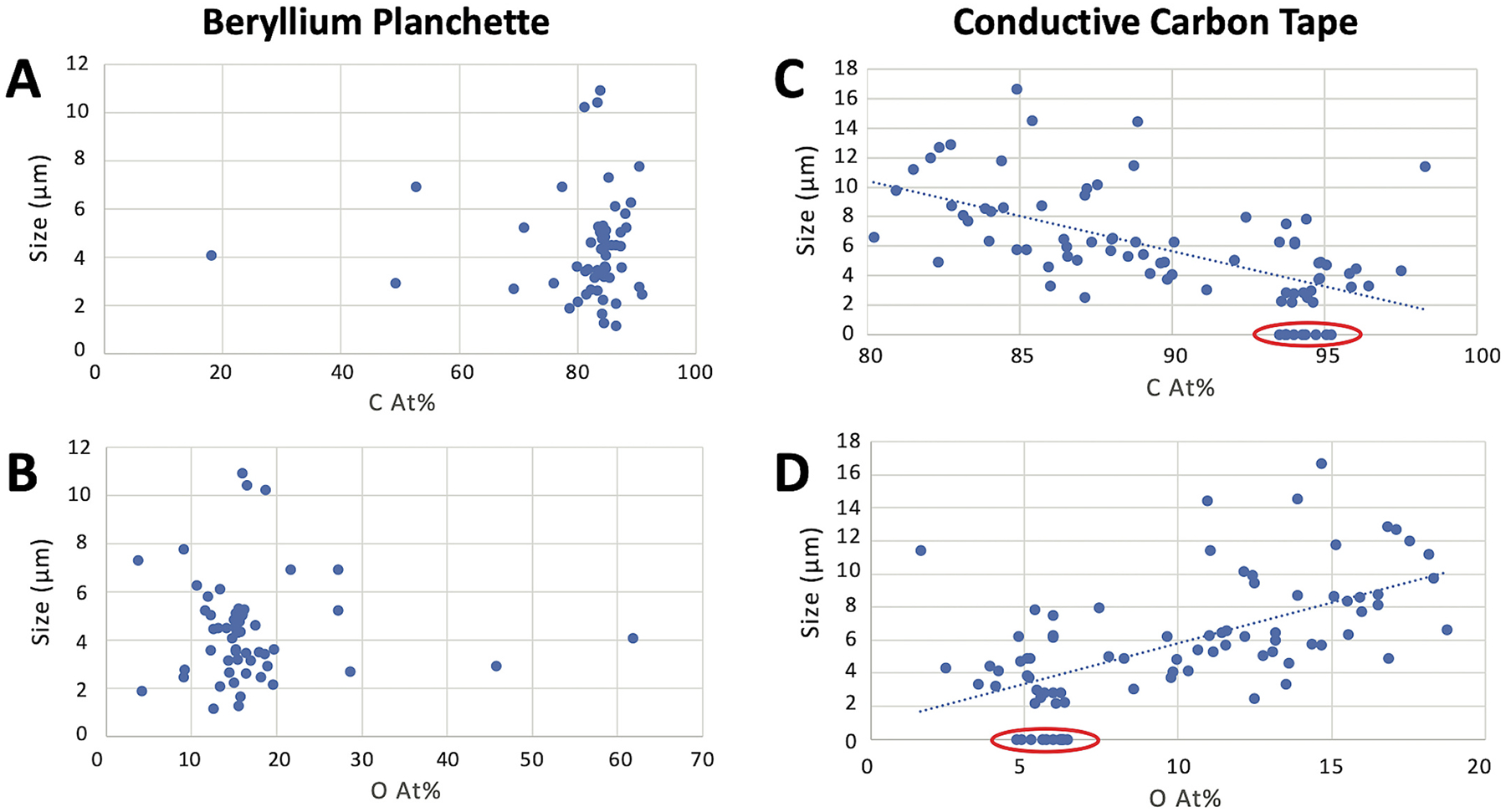
Carbon and oxygen content analyses of National Institute of Standards and Technology (NIST) subbituminous coal standard reference material (SRM 2682c) on a beryllium planchette (A and B) and conductive carbon tape (C and D). Carbon analyses shown in panels A and C. Oxygen analyses shown in panels B and D. Analyses of conductive carbon tape circled in red. *Note*. At.% = atomic weight percent. C = carbon. O = oxygen.

Through analysis of NIST SRM on the beryllium planchette, we found an average carbon content of 82 ± 3 (2σ error) At.% and oxygen content of 17 ± 2 At.%, averaged across all measured particles (Fig. 3). When analyzed on carbon tape, larger NIST SRM particles had average carbon content of 87 ± 1.4 At.% and oxygen content of 13 ± 1.2 At.%, somewhat higher in carbon and lower in oxygen than NIST on the beryllium planchette (Fig. 3). Smaller particles (< 5 μm) had an average carbon and oxygen content of 93 ± 1.4 and 7 ± 1.3 At.%, respectively, which was significantly different from small particles of NIST SRM on the beryllium planchette (Fig. 3). Analyses of conductive carbon tape are circled in red (Fig. 3). This demonstrates the effect of increasing influence of the carbon tape background upon measured carbon and oxygen content as particle size decreases. For small coal particles, there is a possibility of electron penetration through the particle into the carbon tape substrate, or the electron beam interaction volume can surpass the particle edge, influencing measurements of carbon and oxygen composition via EDX.

**Fig. 4.**
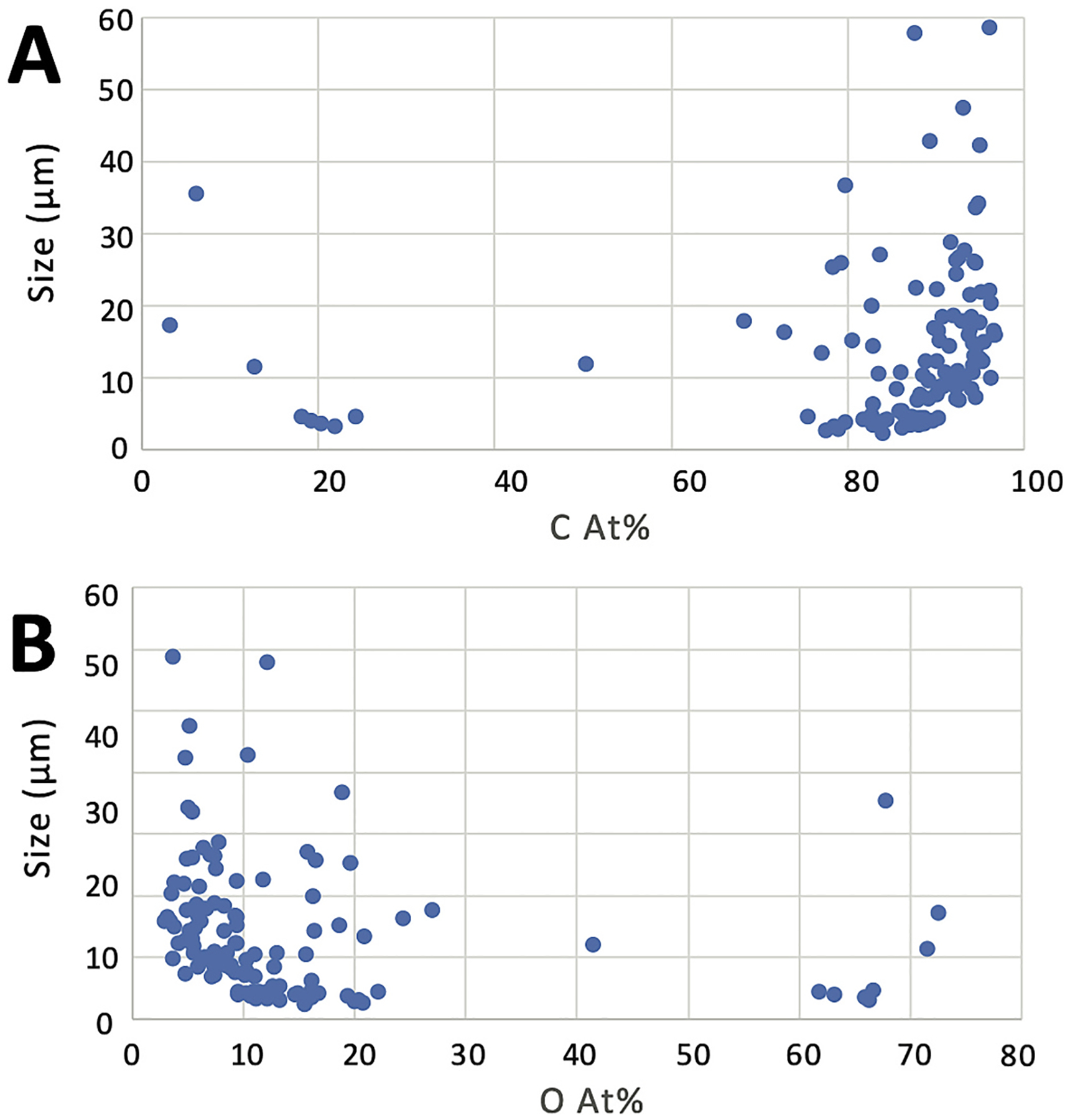
Carbon (A) and oxygen (B) content analyses of positive control coal material from the open-air coal terminal in Curtis Bay, Baltimore, Maryland, USA on a beryllium planchette. *Note*. At.% = atomic weight percent. C = carbon. O = oxygen.

Analysis of positive control coal material from the open-air coal terminal in Curtis Bay on the beryllium planchette yielded two groups of particles: (1) those with carbon = 75–97 At.% and oxygen = 3–20 At.% (average carbon = 83 ± 3.9 At.%, oxygen = 14 ± 2.8 At.%), and (2) those with much lower carbon and higher oxygen content. This second group represents analyses of inorganic minerals. There was no strong compositional trend with particle size observed (Fig. 4).

**Fig. 5.**
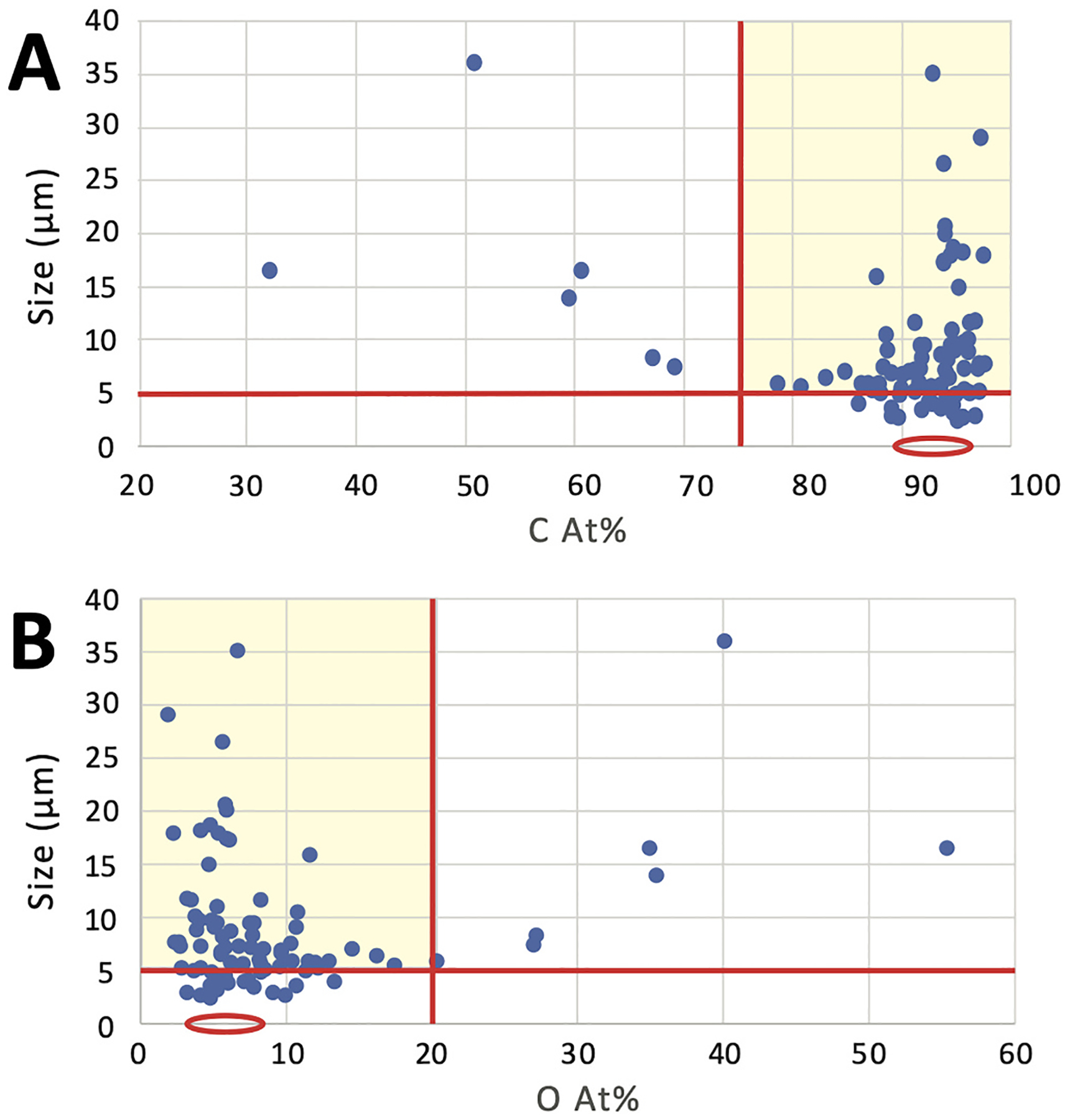
Carbon (A) and oxygen (B) content analyses of positive control material coal from the open-air coal terminal in Curtis Bay, Baltimore, Maryland, USA on conductive carbon tape. Particle size (longest visible dimension >5 μm) and elemental compositional (carbon ≥75 At.% and oxygen ≤20 At.%) limits shown by red lines bounding the yellow bow of accepted coal composition and size for the coal dust classification criteria. Red circles represent analyses of the carbon tape substrate. *Note*. At.% = atomic weight percent. C = carbon. O = oxygen.

Analyses of coal terminal positive control coal material on conductive carbon tape also yielded no clear trend of carbon and oxygen composition with size (Fig. 5). However, the average L < 5 μm particle had carbon = 93 ± 1.6 At.% and oxygen = 7 ± 1.5 At.%. Larger coal particles (L > 5 μm) had a wider range of carbon (average: 89 ± 2.7 At. %) and oxygen (10 ± 2.2 At.%) compositions, but most were in the range of carbon = 88–97 At.% and oxygen = 3–11 At.% (Fig. 5).

A Monte Carlo simulation of electron scattering of NIST bituminous coal SRM resulted in electron penetration depth of approximately 2 μm at 15 kV accelerating voltage (Fig. S1). These findings also informed our decision to restrict SEM-EDX particle analysis on conductive carbon tape to particles of L > 5 μm. Even though the Monte Carlo simulations indicate L = 3 μm may be sufficient (Fig. S1), we selected a more conservative size cutoff since coal particles can have length dimensions longer than thickness. Coal particles with L < 5 μm may have been collected on field samples and yet excluded from analysis by this size cutoff; therefore, the number of coal particles identified is likely a conservative estimate.

On both the beryllium planchette and carbon tape, the average measured carbon and oxygen content by At.% of coal particles were similar. The comparative analysis of positive control coal material sourced from the coal terminal determined carbon ≥75 At.% and oxygen ≤20 At.% as coal dust compositional limits (red lines and yellow box in Fig. 5). Adhering to these compositional limits, a minor fraction (approximately 7 %) of positive control coal particles were eliminated from being considered coal. The compositional limits also align with relevant literature setting coal compositional criteria for SEM-EDX analysis (Sarver et al., 2021; Sellaro et al., 2015). These limits also acknowledge the observed and well-documented heterogeneous nature of coal dust, containing carbonaceous particles, silicates, minerals, soil, and other particle types (Raju and Rom, 1998). The selected elemental compositional limits aim to optimize the selection of carbonaceous coal particles and exclude non-coal particles that were present in standard reference and positive control material, as well as expected in field samples. Therefore, these elemental compositional criteria conservatively identify coal dust particles, minimizing false positive identification of coal particles in field samples.

##### 2.3.2.2. Particle morphology.

**Fig. 6.**
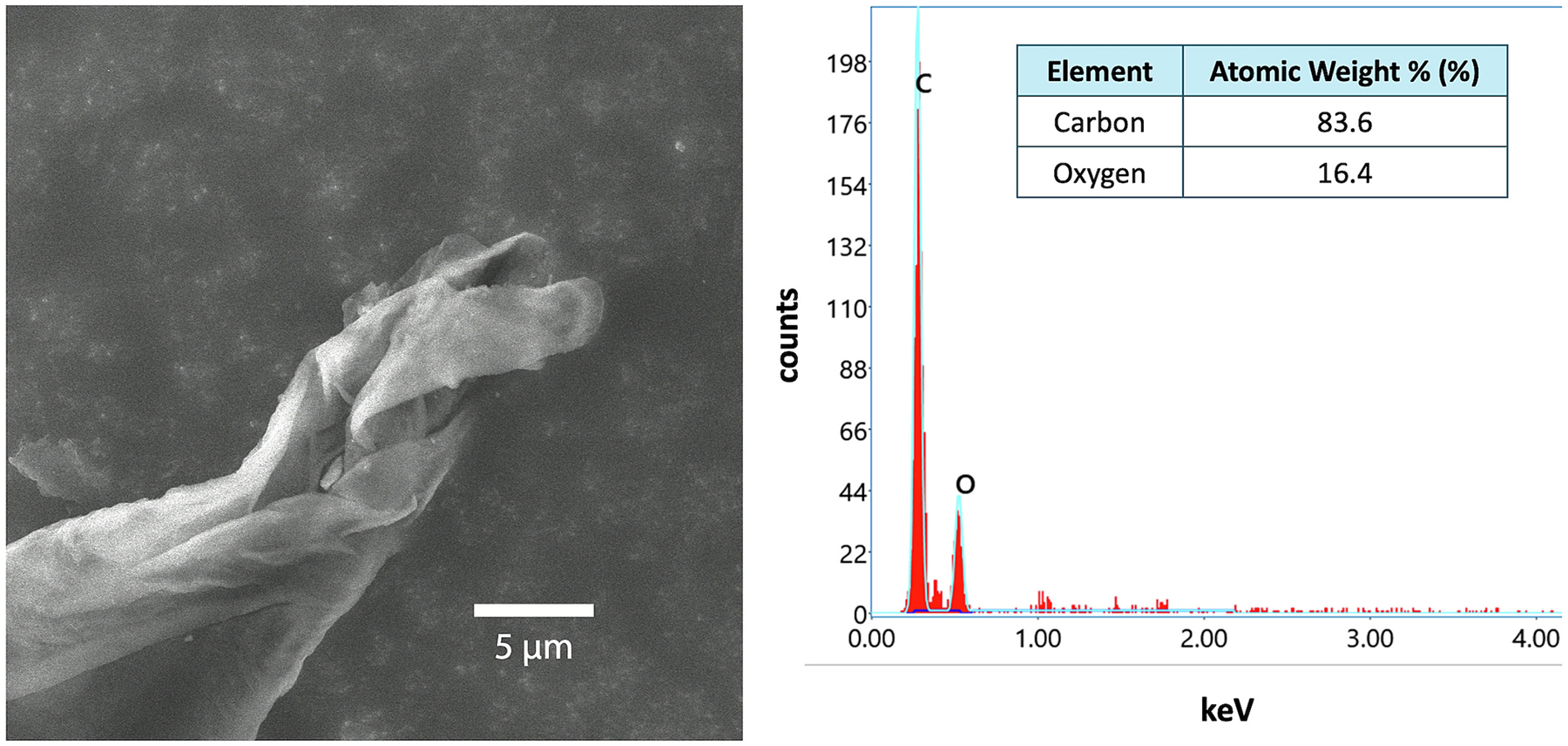
High magnification image (secondary electrons) (left) and elemental composition (right) of a fibrous particle observed on a field sample. Measured elemental composition was consistent with expected coal dust composition, however, morphology differed from expected mineral-like morphology of coal dust. *Note*. The 5 μm scale bar in the coal particle image. keV = kilo-electron-volts.

Consistent with relevant literature (Sellaro et al., 2015; Kamanzi et al., 2024; Rawat and Yadav, 2020; Zazouli et al., 2021; LaBranche et al., 2021), coal particles in both positive coal materials were found to have a mineral-like morphology (e.g., angular and rough, not spherical or fibrous) (Figs. S2 and S3). In the manual analytical protocol described herein, we quantitatively assessed the coal particle morphology to distinguish particles with equivalent elemental composition to that of the coal particles, but clearly reflect the morphology of non-coal particles (Fig. 6). For example, we observed a fibrous particle on a field sample that, when analyzed via EDX, possessed elemental composition consistent with the size limit and coal particle compositional criteria (carbon = 84 At.%, oxygen = 16 At.%; Fig. 6). However, the fibrous morphology greatly differs from expected mineral-like coal particle morphology (Fig. 6); therefore, this particle was excluded from identification as a coal particle.

In summary, the size limit (L > 5 μm), primary elemental compositional limits (carbon ≥75 At.%, oxygen ≤20 At.%), and mineral-like morphology were determined to identify coal particles in this analysis of passive settled dust collected on carbon conductive tape.

### 2.4. SEM-EDX procedure to analyze settled dust samples

#### 2.4.1. Sample preparation

At a cleaned workspace, free from any positive control material, sections of conductive carbon tape (approximately 8 × 8 mm^2^) were cut from field collection, laboratory blank, or field blank samples and mounted on an aluminum SEM stub (Ted Pella, Inc., Redding, CA, US). Mounted samples were stored in sealed and labeled individual stub mailing tubes (Ted Pella, Inc., Redding, CA, US) when not under the SEM. All samples were handled with gloves and cleaned tools. Samples were not coated prior to SEM-EDX analysis.

#### 2.4.2. Particle selection and SEM-EDX analysis

To begin the analysis, the SEM operator saves nine area positions at pre-specified locations focused at a magnification of 33× (Fig. S4). SEM-EDX operating parameters used are presented in Table 1. At each area position, focused images at magnifications of 250× and 500× are captured. In each area position at a magnification of 500 X, individual particles are selected in an anti-clockwise fashion from the center of the field of view (FOV) (Fig. S5). A group of ten particles (L > 5 μm) are selected, then analyzed via EDX.

Coal particles were identified using the classification criteria described in Section 2.3.2. For selected particles identified as coal dust, the SEM operator notes the particle number, area position number, particle L in μm, and particle morphology, then captures a higher magnification image of the coal particle.

If no coal particle is identified in a group of ten, the next ten particles in the FOV are selected, continuing in an anti-clockwise fashion. Once all particles (L > 5 μm) in the 500 x FOV have been selected and analyzed, the operator repeats the preceding steps in the next saved area position.

Analysis of each sample stops when (1) at least five coal particles are identified or (2) 100 total particles (regardless of type) are analyzed–-whichever stop limit is reached first. If these stop limits are not reached after analyzing particles in the first nine area positions, the operator saves a new set of nine area positions in a 33× magnification FOV that does not overlap with the previous FOV. Samples with light particle loading are analyzed using a slightly amended protocol described in Supplementary Text S1.

An operator from the JHU-MCP independently (at the time of analysis and reporting) analyzed laboratory and field blanks and blinded field samples using the SEM-EDX analytical protocol described herein (see Section 2.4).

### 2.5. Data analysis

Each FOV at magnification of 500× used to analyze settled dust particles has an area of 0.066 mm^2^. After reaching a stop limit (see Section 2.4.2), the SEM operator can calculate the number of coal particles identified out of the total number of particles analyzed and total area analyzed (mm^2^). The total number of coal dust particles per mm^2^ analyzed can be used to compare the density or loading of identified coal dust particles between different samples and/or sampling locations.

This protocol and analysis can be used to determine: (1) the presence-absence of coal dust particles in settled dust passively collected on conductive carbon tape; (2) the fraction of coal particles out of the total number of particles analyzed; and (3) approximate loading of settled particles and/or coal particles per mm^2^ analyzed on each sample.

## 3. Results

**Fig. 7.**
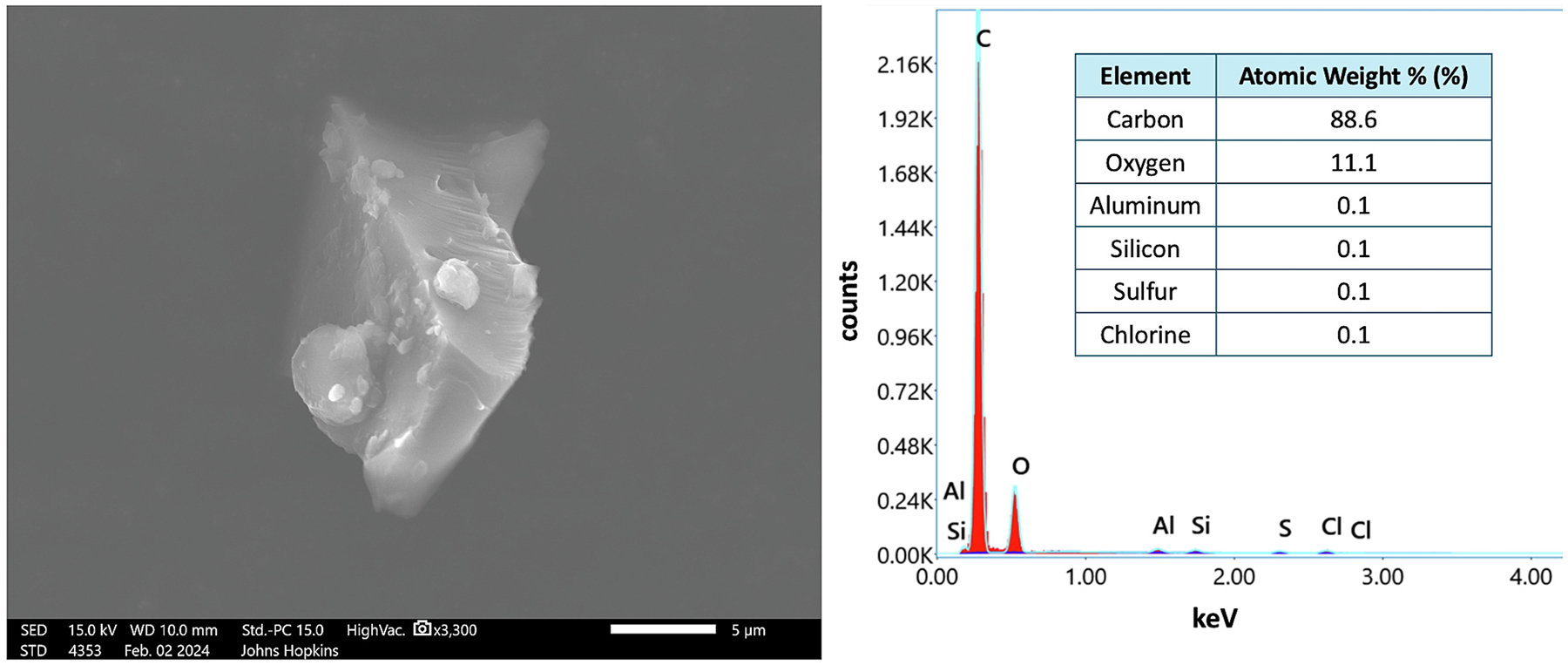
High magnification image (secondary electrons) (left) and elemental composition (right) of one out of seven positively identified coal particles observed at Location A (345 m from coal terminal). This was the 1st particle in the 7th area position analyzed. *Note*. The 5 μm scale bar in the coal particle image. keV = kilo-electron-volts.

On the proximal sample obtained from Location A, 345 m from the coal terminal, eight area positions, equaling an area of 0.53 mm^2^, were required to identify at least five coal particles (Table 2). Across the eight area positions, 82 total particles were analyzed, regardless of type. The approximate total particle loading on this sample was 155 particles/mm^2^ (Table 2). Out of the 82 particles analyzed, seven were positively identified as coal particles (Figs. 7; S6–S10). The high magnification image and elemental composition is shown for one of the seven coal particles (Fig. 7). The fraction of settled coal particles observed was approximately 9 %, and the measured coal dust loading was 13.2 coal particles/mm^2^ (Table 2; Fig. 9).

**Fig. 8.**
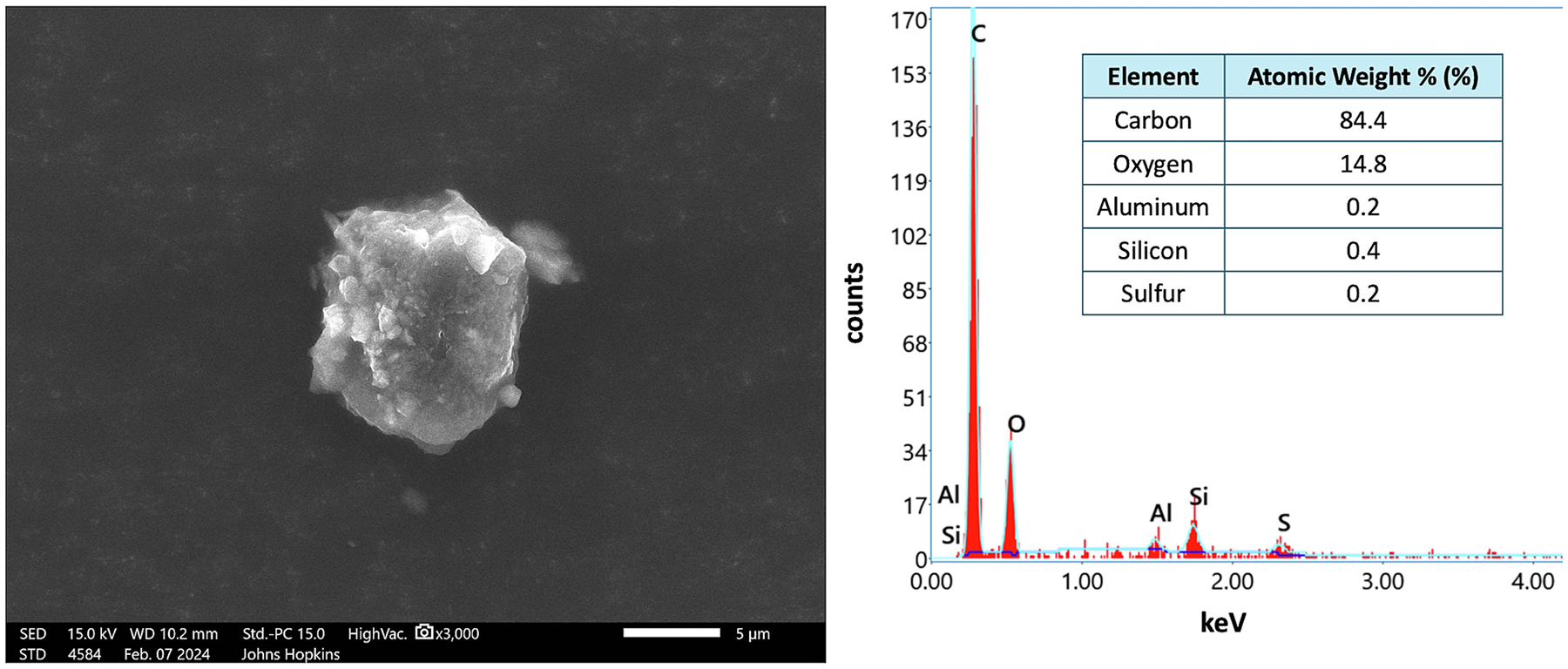
High magnification image (secondary electrons) (left) and elemental composition (right) of one out of six positively identified coal particles observed at Location B (1,235 m from coal terminal). This was the 1st particle in the 5th area position analyzed. *Note*. The 5 μm scale bar in the coal particle image. keV = kilo-electron-volts.

**Fig. 9.**
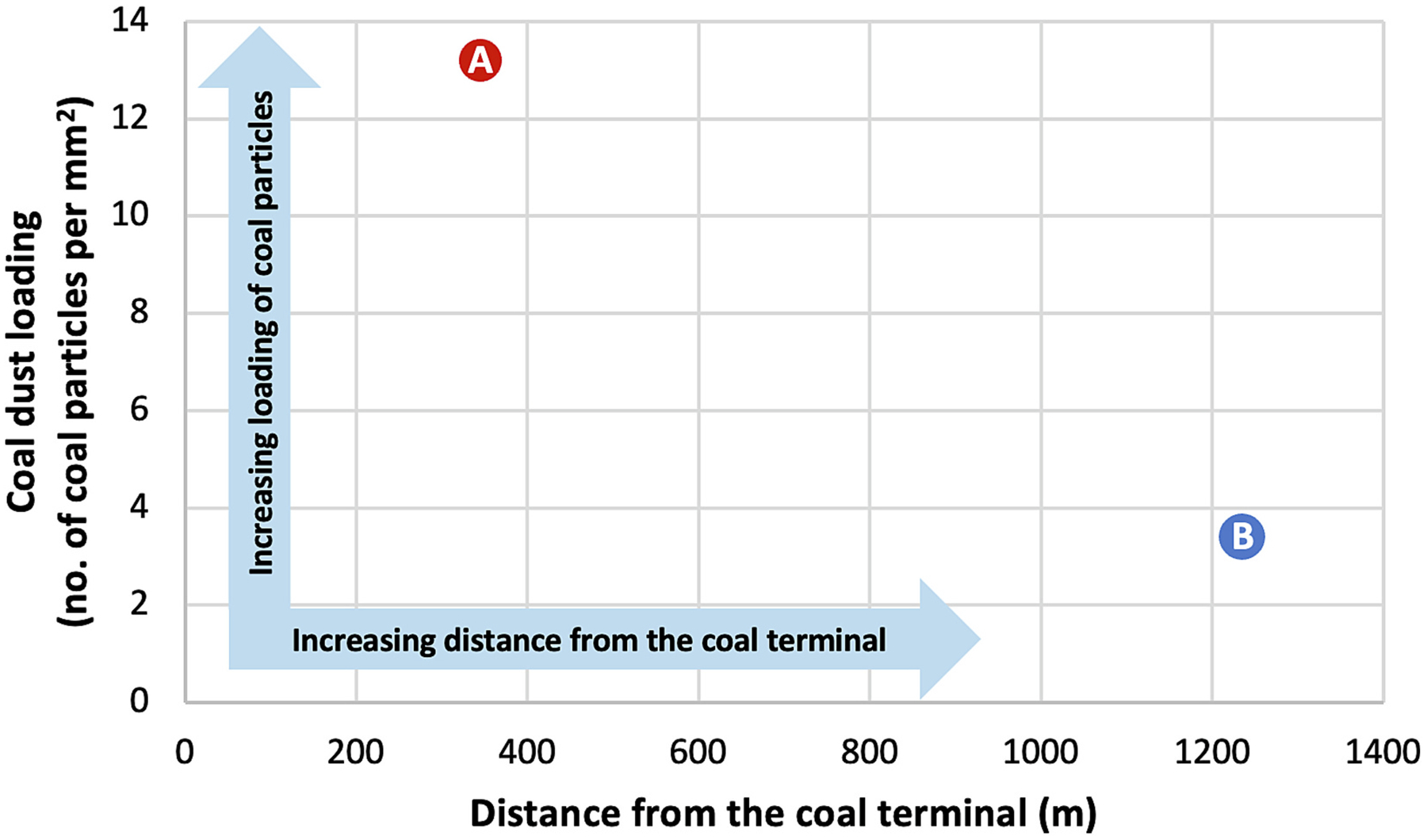
Coal dust loading observed on each settled dust sample collected passively over a 3-day sampling period (October 26–29, 2023) in Curtis Bay, Maryland, USA. Coal dust loading of 13.2 coal particles/mm^2^ was observed at Location A, 345 m from the coal terminal (red circle). Coal dust loading of 3.4 coal particles/mm^2^ was observed at Location B, 1235 m from the coal terminal (blue circle).

On the distal sample obtained from Location B, 1235 m from the coal terminal, 27 area positions, equaling an area of 1.78 mm^2^, were required to identify at least five coal particles (Table 2). Across the 27 area positions, 78 total particles were analyzed, regardless of type (Table 2). The approximate total particle loading on this sample was 44 particles/mm^2^ (Table 2). Out of the 78 particles analyzed, six were positively identified as coal particles (Figs. 8; S11–S15). The high magnification image and elemental composition is shown for one of the six coal particles (Fig. 8). The fraction of settled coal particles observed was approximately 8 %, and the coal dust loading was 3.4 coal particles/mm^2^ (Table 2; Fig. 9).

No coal particles were identified on the laboratory blank nor field blanks from either sampling location.

## 4. Discussion

Coal particles were identified on settled dust samples proximal (Location A) and distal (Location B) to the open-air coal terminal in the community of Curtis Bay in Baltimore, Maryland, USA. At each sampling location, the fraction of particles identified as coal dust among the total particles characterized was 9 % and 8 %, respectively. To contextualize this finding, Sellaro et al. identified primarily carbonaceous particles–defined as carbon ≥70 At.%; oxygen ≤30 At.%–similar to the elemental compositional criteria employed herein–as comprising an average of 12 %, 25 %, and 40 % of the total particles observed from sampling at a roof bolter, belt drive, and intake locations, respectively, of an underground coal mine in Central Appalachia (Sellaro et al., 2015).

Although our interest was in the identification and characterization of coal dust particles, the sampling and SEM-EDX analytical approach described can be adapted to select and characterize other particle types of interest. For example, some settled dust particles analyzed matched the coal dust elemental composition but failed to be morphologically characterized (e.g., fibers, round soot spheres, and pollen). Steel, aluminum, and other non‑carbonaceous particles were also observed on settled dust samples, reflective of the various industrial operations present in Curtis Bay.

This study had several strengths. First, as community-driven research, the sampling and analytical methods were responsive to and guided by Curtis Bay residents’ questions regarding the presence of coal dust in the community. Curtis Bay and South Baltimore community members from the South Baltimore Community Land Trust, Community of Curtis Bay Association, and Benjamin Franklin High School drove research questions and objectives, collaborated with academic partners in pilot testing and field collections, co-wrote this manuscript, and facilitated public dissemination of our findings at community meetings and discussions. Second, the systematic SEM-EDX protocol to determine the presence-absence of coal dust optimizes SEM-instrument time while achieving sufficient coverage of the mounted sample area. Third, the estimated coal dust loading metric can support comparisons between sites relative to local sources of coal dust. Importantly, this study addresses the paucity of information about the presence of coal dust in community settings and the lack of systematic, practical methods to fill this knowledge gap. Additionally, the sampling and analytical methods described herein can be adapted to other particle types or pollution sources of interest in a community setting.

Our study had some limitations. First, using the manual SEM-EDX protocol described, we were unable to determine the presence of L < 5 μm size coal particles (see Section 2.3.2). Other available analytical electron microscopy approaches (e.g., transmission electron microscopy) and automated or computer-controlled SEM-EDX procedures (Johann-Essex et al., 2017a; Johann-Essex et al., 2017b; Sarver et al., 2024) can be used to analyze L < 5 μm size coal particles, even in the nanoparticle size range. This study does confirm the presence of L > 5 μm coal dust and we expect coal dust to contribute to a fraction of ambient and settled fine particulate matter (Ostro et al., 2023b; Junge, 1955). These finer L < 5 μm size particles can travel deeper into the human respiratory tract and cause more severe health impacts than larger coarse particles. However, cytotoxic health effects of coarse size (average D50 = 10.9 ± 1.8 μm) coal particles, driven by physical particle characteristics, have been observed (Kamanzi et al., 2024). Second, it is challenging to produce quantitative data about the total number and precise proportion of settled coal particles using a manual SEM-EDX analysis protocol of settled dust samples. An automated SEM-EDX system with particle identification and analysis capabilities can provide this quantitative data and further information about individual particle composition and structure (Kamanzi et al., 2024; Johann-Essex et al., 2017a; Kamanzi et al., 2022). These systems require specialized–at times proprietary–software and prerequisite reference lists for particle compositional characterization.

Despite these limitations, this study confirms the presence of coal dust in settled dust samples collected from residential areas of Curtis Bay. Future studies could use auto-SEM-EDX systems to obtain quantitative data and assess the concentration, flux, and spatiotemporal variability of coal dust accumulation in the Curtis Bay community using both passive (Wagner et al., 2001; Leith et al., 2007) and active aerosol sampling methods. Further consideration of particle fate and transport and environmental conditions with inclusion of background sampling sites outside of Curtis Bay could assist with coal dust source identification in the community setting. Future research can use the coal dust loading metric and particle counts to establish a statistical comparison in the community-level deposition of coal particles varying by distance from suspected sources and sampling duration. In this study, the coal dust loading sampled further from the coal terminal appeared to be lower than the coal dust loading at the proximal sampling site, however, we are unable to establish a statistically significant difference.

## 5. Conclusion

This study involved the development and application of an SEM-EDX protocol to systematically characterize the presence-absence of coal dust in passive settled dust samples collected in a residential community bordering a coal terminal. We confirmed the presence of coal dust in residential areas of the Curtis Bay community at sampling locations proximal to (345 m) and distal from (1235 m) an open-air coal terminal. These findings respond to decades-long concerns of Curtis Bay community members about coal dust presence in their community. The approach described herein may have practical utility for other communities neighboring coal terminals to collect settled dust samples and generate information about the presence of coal dust using reproducible and standardized methods for particle analysis.

## Supplementary Material

Supplementary Material

## Figures and Tables

**Table 1:** Scanning electron microscope (SEM) and energy dispersive X-ray spectrometer (EDS) operating parameters used.

	SEM		EDS
Electron mode		Secondary electrons	
Accelerating voltage		15 kV	
Beam current		15 nA	
Vacuum mode		High vacuum	
Working distance		10 mm	
Collection time	–		10 s
Brightness/contrast		Adjust as needed	

**Table 2:** Summary of findings from analysis of settled dust samples collected passively over a 3-day sampling period (October 26–29, 2023) in Curtis Bay, Maryland, USA.

		Total			Coal-specific		
Distance from coal terminal (m)	Number of area positions analyzed^a^	Total area of sample analyzed (mm^2^)^b^	Total number of particles analyzed	Total particle loading (pt/mm^2^)^c^	Number of coal particles identified	Fraction of coal particles (%)^d^	Coal dust loading (coal pt/mm^2^)^e,f^
345	8	0.53	82	155	7	9	13.2
1235	27	1.78	78	44	6	8	3.4

a Number of area positions required to reach an analytical stop limit (i.e., five coal particles identified or 100 total particles analyzed).

b Each 500× magnification field of view was 0.066 mm^2^ area.

c Pt = number of particles.

d Number of coal particles identified divided by the total number of particles analyzed.

e Coal pt = number of coal particles.

f Number of coal particles identified divided by the total area of the sample analyzed.

## Data Availability

De-identified data will be made available upon request.

## HIGHLIGHTS

- Coal terminal neighbors in Curtis Bay, MD raised concerns about coal dust exposure..
- Settled dust sampled in 2 residential areas up to 1235 m from coal terminal
- SEM-EDX analysis confirmed presence of coal dust in both residential samples.
- Provides evidence of coal dust presence in a community neighboring coal terminal

## Abbreviations:

US: United States
PM_2.5_: Particles of aerodynamic diameter ≤ 2.5 μm
MDE: Maryland Department of the Environment
BSPH: Johns Hopkins Bloomberg School of Public Health
JHU-MCP: Johns Hopkins University Materials Characterization and Processing Facility
UMD: University of Maryland
SEM-EDX: Scanning electron microscopy and energy dispersive X-ray spectroscopy
NIST: National Institute of Standards and Technology
SRM: Standard reference material
L: Length of longest visible dimension
At.%: Atomic weight percent
FOV: Field of view
