# Supplementary material for: Use of electron microscopy to determine presence of coal dust in a neighborhood bordering an open-air coal terminal in Curtis Bay, Baltimore, Maryland, USA
Source: Sci Total Environ. Author manuscript; Available in PMC 2025 Jan 3. (PMC11698221; doi:10.1016/j.scitotenv.2024.176842)

***Supplementary Material***

**Use of electron microscopy to determine presence of coal dust in a neighborhood bordering an open-air coal terminal in Curtis Bay, Baltimore, Maryland, USA**

Matthew A. Aubourg^a,b*^, Kenneth J.T. Livi^c^, Gregory G. Sawtell^d,e^, Carlos C. Sanchez-Gonzalez^e^, Nicholas J. Spada^f^, Russell R. Dickerson^g^, Wen-an Chiou^h^, Conchita Kamanzi^i,j^, Gurumurthy Ramachandran^b^, Ana M. Rule^b^, Christopher D. Heaney^a,b,k,l^

^a^Community Science and Innovation for Environmental Justice (CSI EJ) Initiative, Department of Environmental Health and Engineering, Johns Hopkins Bloomberg School of Public Health, Baltimore, Maryland, 21205, United States; [maubour1@jh.edu](mailto:maubour1@jh.edu), [cheaney1@jhu.edu](mailto:cheaney1@jhu.edu)

^b^Department of Environmental Health and Engineering, Johns Hopkins Bloomberg School of Public Health, Baltimore, Maryland, 21205, United States; [maubour1@jh.edu](mailto:maubour1@jh.edu), [gramach5@jhu.edu](mailto:gramach5@jhu.edu), [arule1@jhu.edu](mailto:arule1@jhu.edu), [cheaney1@jhu.edu](mailto:cheaney1@jhu.edu)

^c^Materials Characterization and Processing, Department of Materials Science and Engineering, Johns Hopkins University, Baltimore, Maryland, 21211, United States; [klivi@jhu.edu](mailto:klivi@jhu.edu)

^d^Community of Curtis Bay Association, Baltimore, Maryland, 21226, United States; [greg@sbclt.org](mailto:greg@sbclt.org)

^e^South Baltimore Community Land Trust, Baltimore, Maryland, 21225, United States; [greg@sbclt.org](mailto:greg@sbclt.org), [carlos@sbclt.org](mailto:carlos@sbclt.org)

^f^Air Quality Research Center, University of California, Davis, California, 95616, United States; [njspada@ucdavis.edu](mailto:njspada@ucdavis.edu)

^g^Department of Atmospheric and Oceanic Science, University of Maryland, College Park, Maryland, 20742, United States; [rrd@umd.edu](mailto:rrd@umd.edu)

^h^Advanced Imaging and Microscopy Laboratory (AIM Lab), Maryland NanoCenter, University of Maryland, College Park, MD, 20742, United States; [wachiou@umd.edu](mailto:wachiou@umd.edu)

^i^Department of Chemical Engineering, Minerals to Metals Initiative, University of Cape Town, Cape Town, 7701, South Africa; [Conchita.Kamanzi@uct.ac.za](mailto:Conchita.Kamanzi@uct.ac.za)

^j^Institute of Infectious Diseases and Molecular Medicine, University of Cape Town, 7925, South Africa; [Conchita.Kamanzi@uct.ac.za](mailto:Conchita.Kamanzi@uct.ac.za)

^k^Department of Epidemiology, Johns Hopkins Bloomberg School of Public Health, Baltimore, Maryland, 21205, United States; [cheaney1@jhu.edu](mailto:cheaney1@jhu.edu)

^l^Department of International Health, Johns Hopkins Bloomberg School of Public Health, Baltimore, Maryland, 21205, United States; [cheaney1@jhu.edu](mailto:cheaney1@jhu.edu)

***Correspondence**: Matthew A. Aubourg; Department of Environmental Health and Engineering, Johns Hopkins Bloomberg School of Public Health, Baltimore, Maryland, US; Email address: [maubour1@jh.edu](mailto:maubour1@jh.edu)

***SUPPLEMENTARY METHODS***

**Supplementary Figure S1.** Monte Carlo simulation of electron-electron scattering in National Institute of Standards and Technology (NIST) bituminous coal standard reference material (NIST certified SRM 8499). Simulations were performed using CASINO (monte CArlo Simulation of electroN trajectory in sOlids, Drouin et. al., 2007), version 2.4.8.1 using default settings and 5,000 simulated electron trajectories from 5 to 20 KeV in 5 KeV increments. Idealized assumptions of the model include spherical coal particles, homogenous chemistry of each particle, and particle chemistry conformed to the NIST-reported major elements (carbon, iron, silicon, sulfur, aluminum, and potassium). Conducted at University of California, Davis Advanced Materials Characterization and Testing (AMCaT) Laboratory. Note: Each simulation is presented at different x- and y-axis scales.


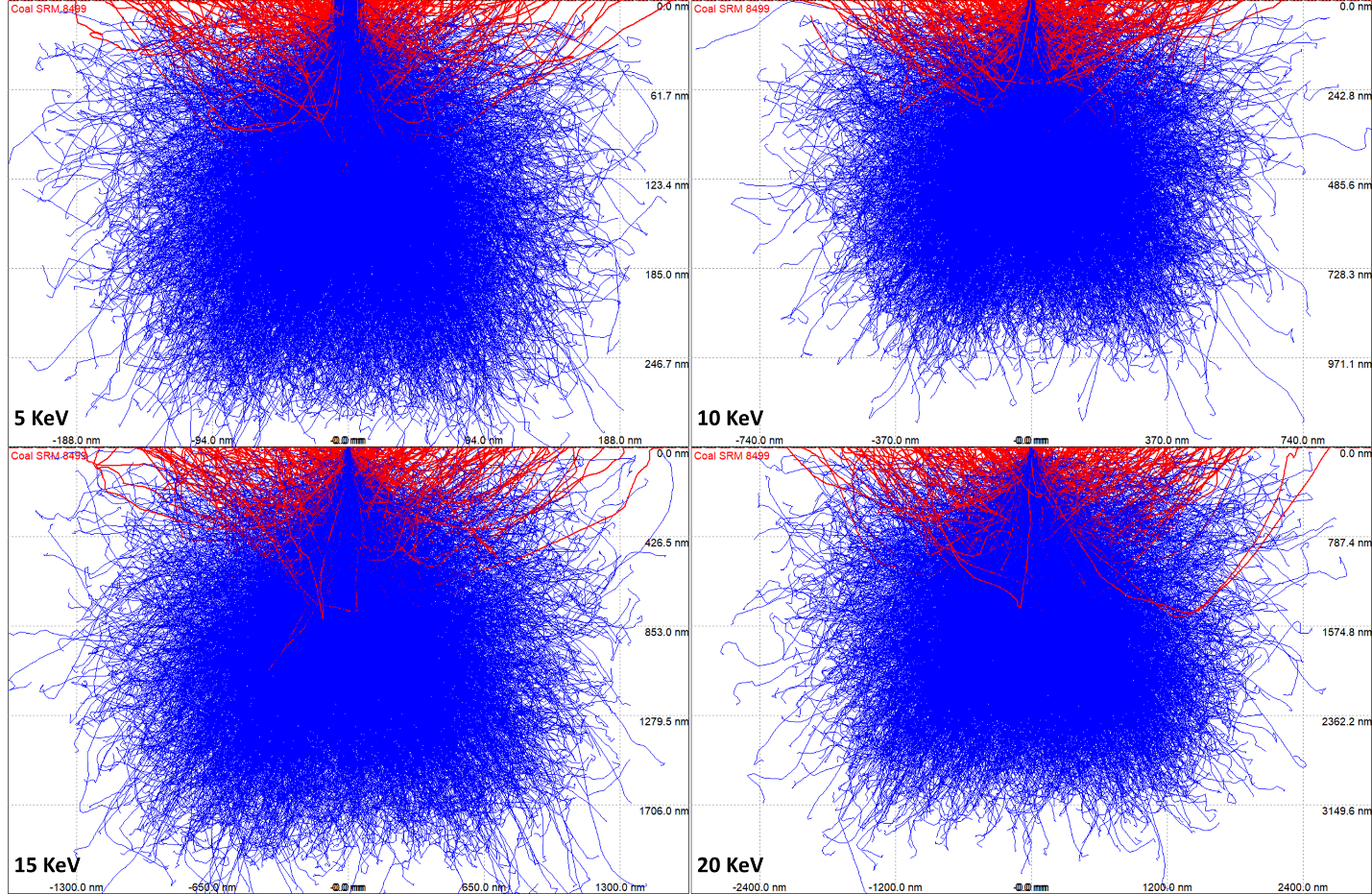

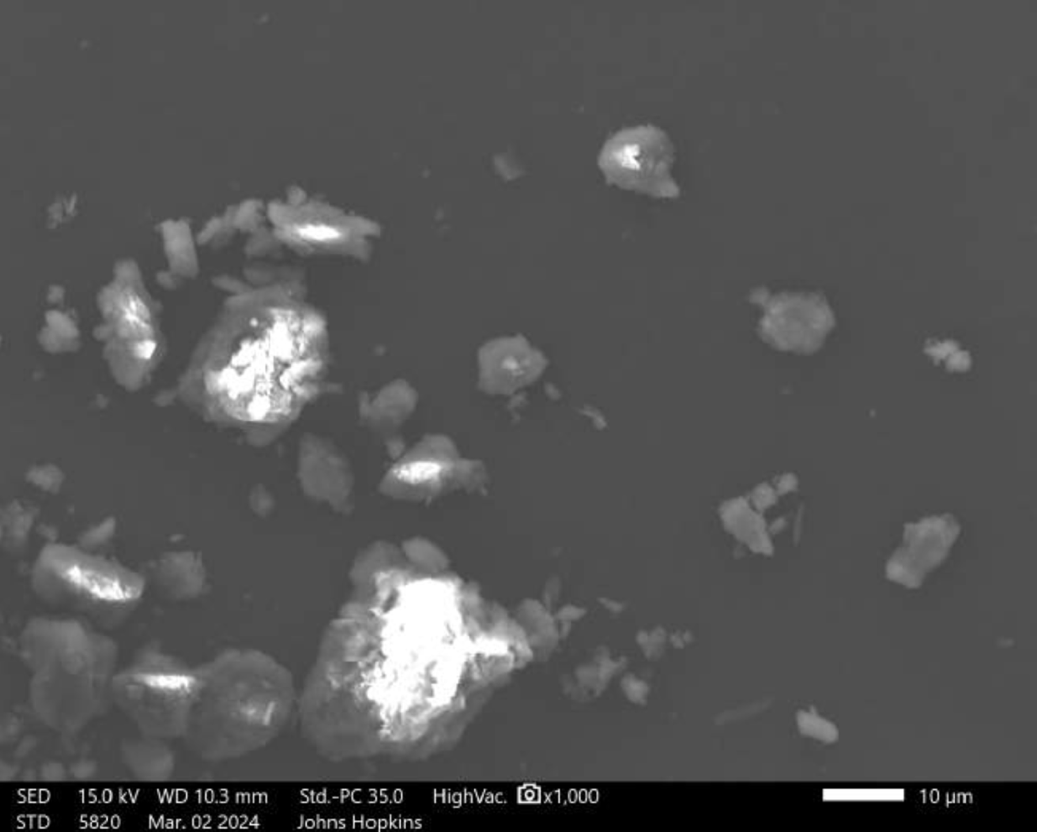


**Supplementary Figure S2.** High magnification image depicting typical particle morphology of National Institute of Standards and Technology (NIST) subbituminous coal standard reference material (SRM 2682c) on a beryllium planchette.


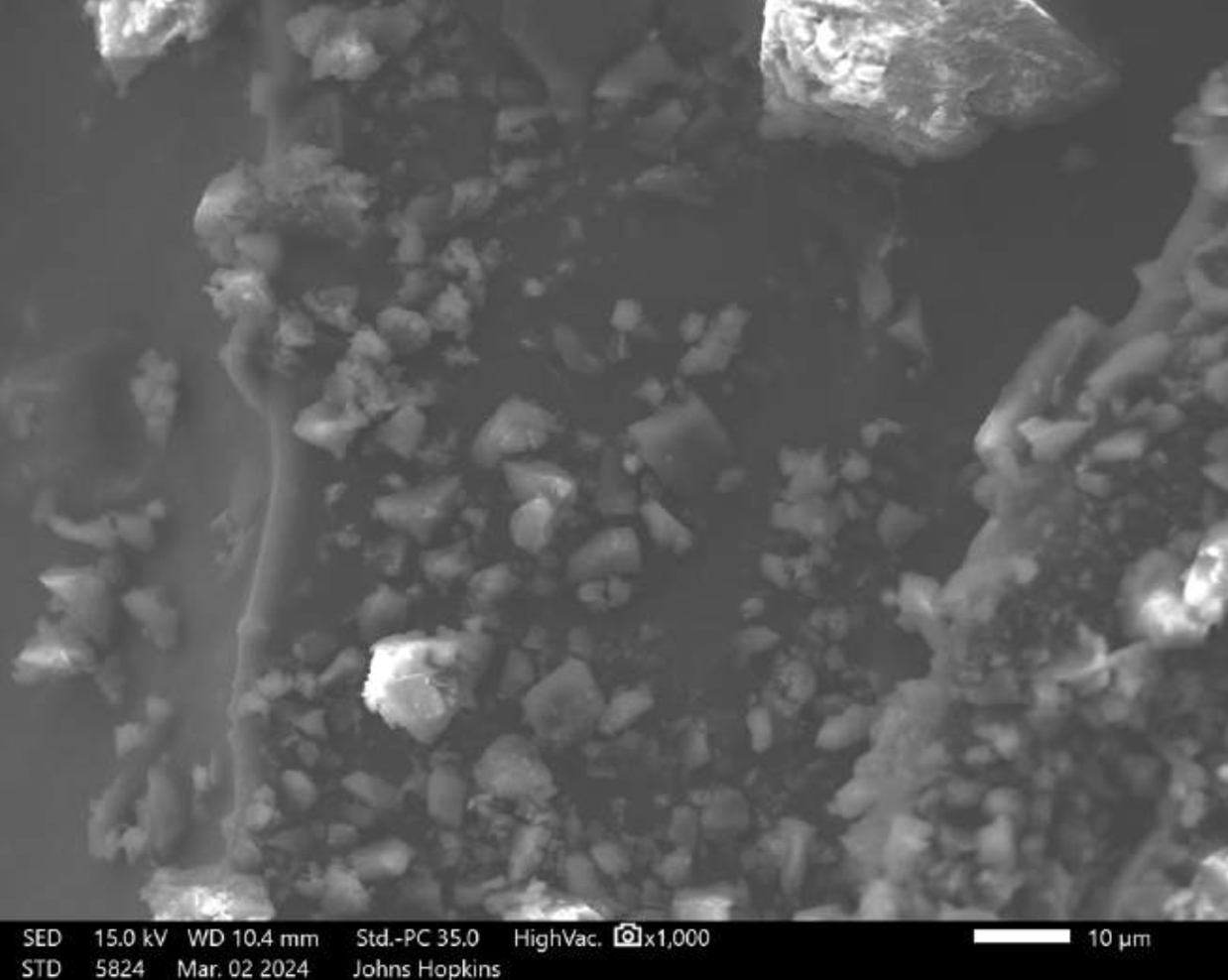


**Supplementary Figure S3.** High magnification image depicting typical particle morphology of positive control coal material from the open-air coal terminal in Curtis Bay, Baltimore, Maryland, USA on conductive carbon tape. This positive control material contained a large fraction of large coarse particles and was lightly ground prior to SEM-EDX analysis.


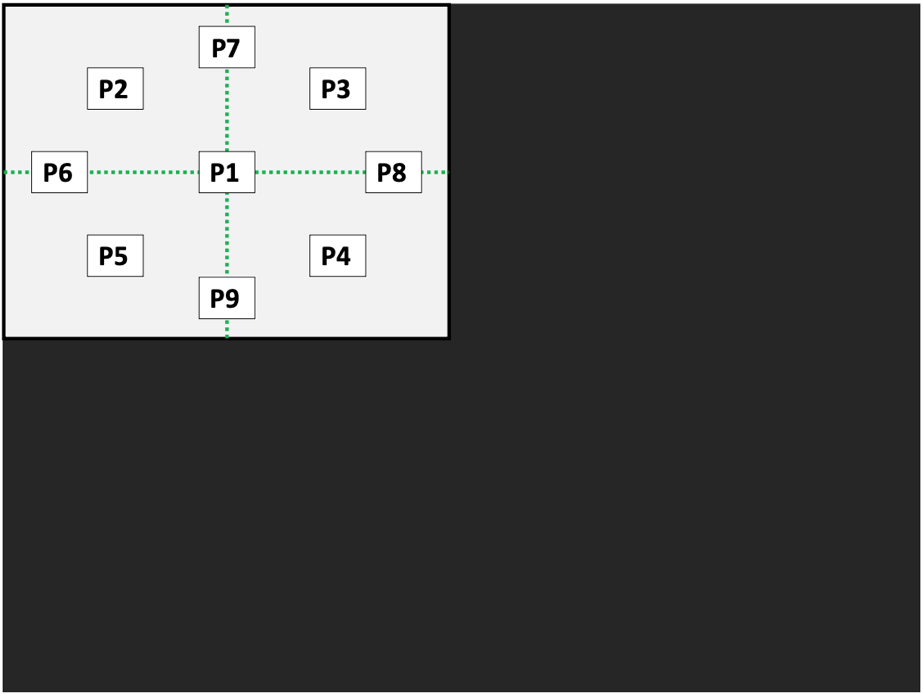


**Supplementary Figure S4.** The first group of nine pre-specified and saved positions, selected at 33x magnification and numbered from P1 to P9, on the sample where analysis will occur in numerical order. Field of view (light grey) and conductive carbon tape (dark grey) are approximately to scale.

**Supplementary Figure S5.** Anti-clockwise selection of ten particles at a time in each area position focused at a magnification of 500x. All particles in the field of view (longest visible dimension > 5 µm) are selected and analyzed.

**Text S1. SEM-EDX analysis of lightly loaded samples**

This SEM-EDX protocol differentiates between moderately to highly versus lightly particle-loaded field samples. The main text describes the analytical protocol for highly to moderately loaded samples, representative of field samples from the Curtis Bay community. For samples lightly loaded with settled dust particles, the operator saves nine area positions at 33 x magnification, identical to the intial steps for the protocol for moderately to heavily loaded samples. At each saved position, two focused images are captured at 250 x and 500 x magnification. At 500 x magnification, all visible particles (L > 5 µm) are selected and analyzed via EDX. Analysis ceases when either (1) five coal particles are postively identified or (2) all particles across 36 area positions (at three non-overlapping 33x magnification fields of view) have been analyzed without identifying five coal particles.

***SUPPLEMENTARY RESULTS***

**Text S3. POSITVELY IDENTIFIED COAL PARTICLES OBSERVED AT LOCATION A**

Location A was the most proximal sampling location to the open-air coal terminal in Curtis Bay at 345 m from the facility. Seven coal particles were postively identified on the sample collected at this location. See main text Figure 3 and Supplementary Figures S10-S14 for all coal particle images and corresponding EDX spectra.

**Supplementary Figure S6.** High magnification image (secondary electrons) (left) and elemental composition (right) of one out of seven positively identified coal particles observed at Location A. This was the 7^th^ particle in the 3^rd^ area position analyzed. keV = kilo-electron-volts.


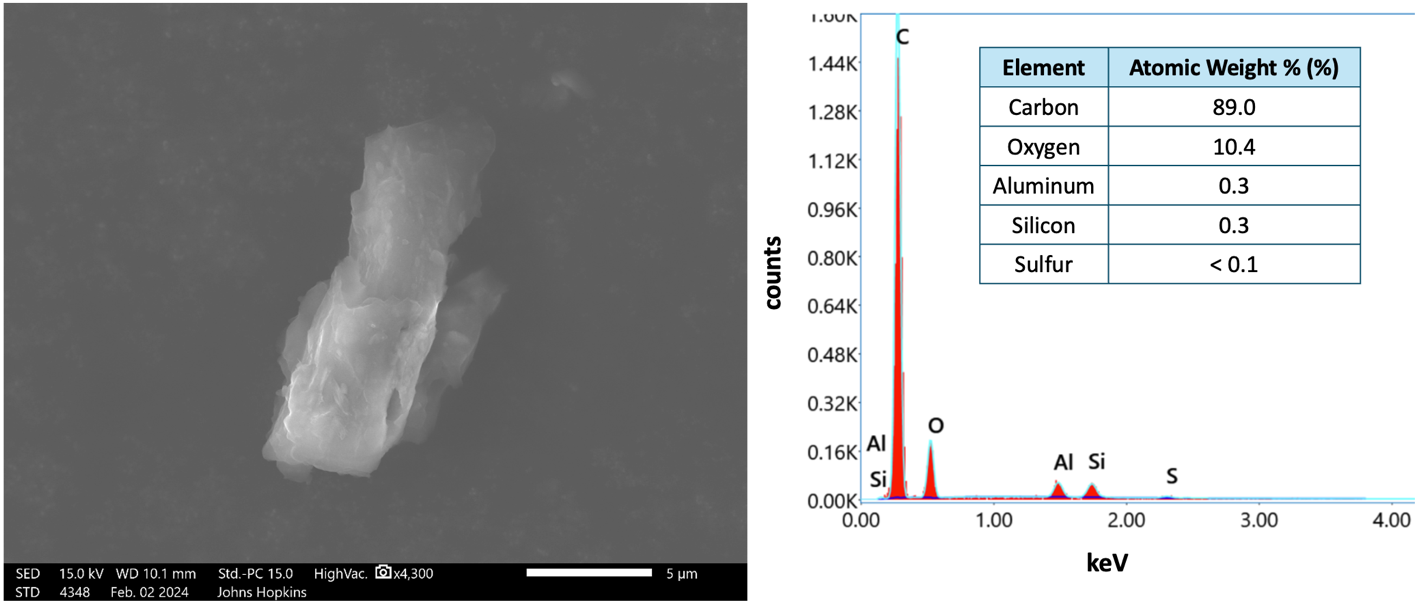


**Supplementary Figure S8.** High magnification image (secondary electrons) (left) and elemental compositions (right) of two out of seven positively identified coal particles observed at Location A. These were the 1^st^ (**A**) and 3^rd^ (**B**) particles analyzed in Position 8.


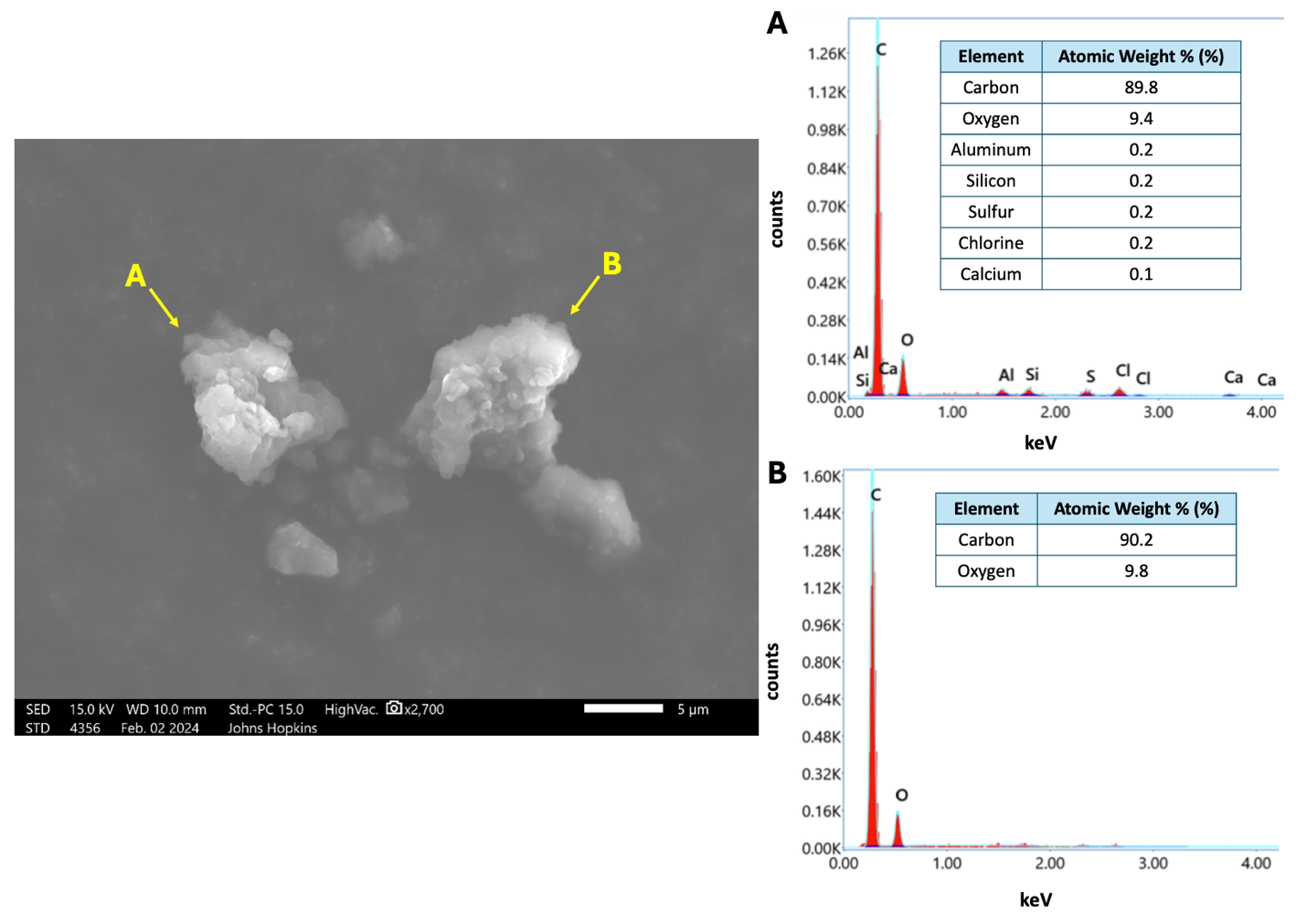


**Supplementary Figure S7.** High magnification image (secondary electrons) (left) and elemental composition (right) of one out of seven positively identified coal particles observed at Location A. This was the 4^th^ particle in the 4^th^ area position analyzed.


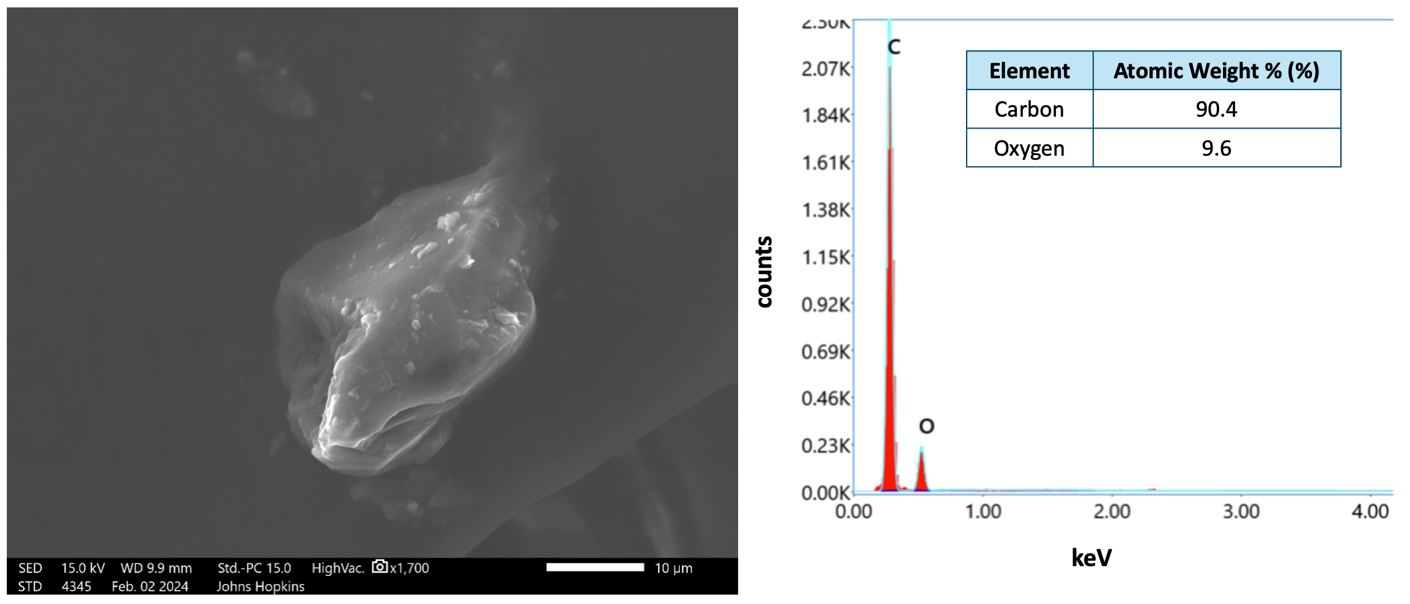


**Supplementary Figure S9.** 500 x magnification image (secondary electrons) (left) and elemental composition (right) of one out of seven positively identified coal particles observed at Location A (red dot). This was the 4^th^ particle analyzed in the 8^th^ area position. High magnification image of this particle is unavailable.


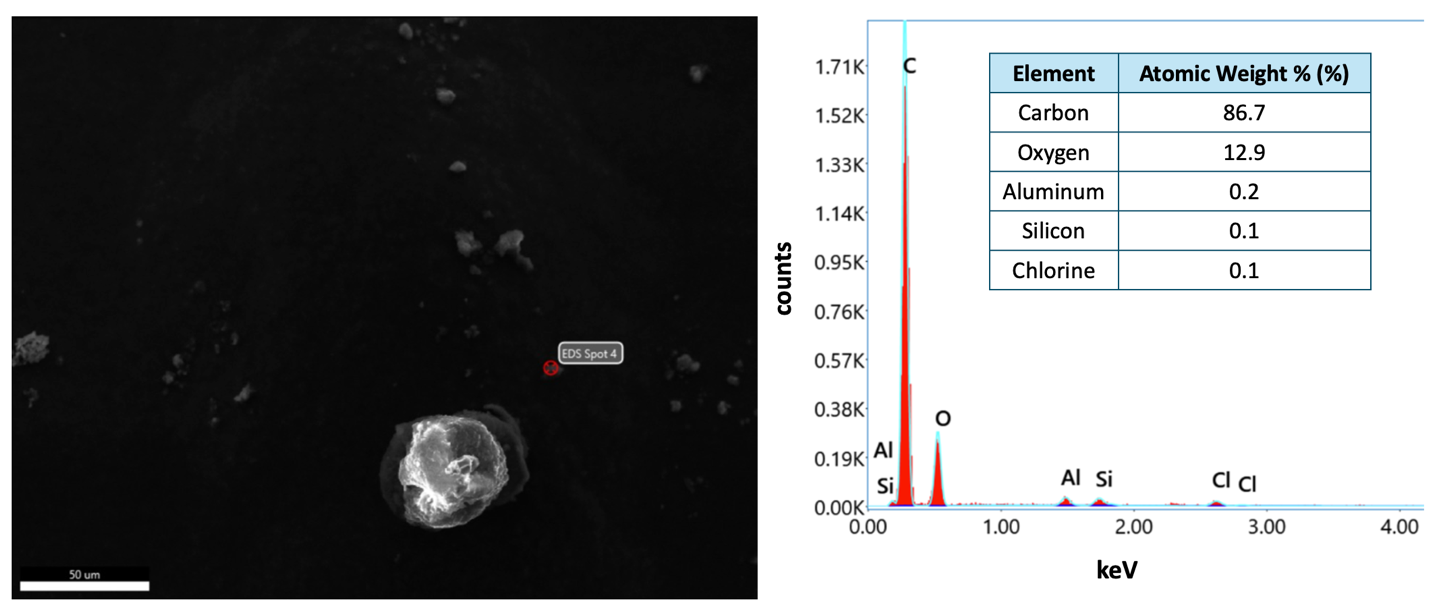


**Supplementary Figure S10.** High magnification image (secondary electrons) (left) and elemental composition (right) of one out of seven positively identified coal particles observed at Location A. This was the 8^th^ particle analyzed in the 8^th^ area position.


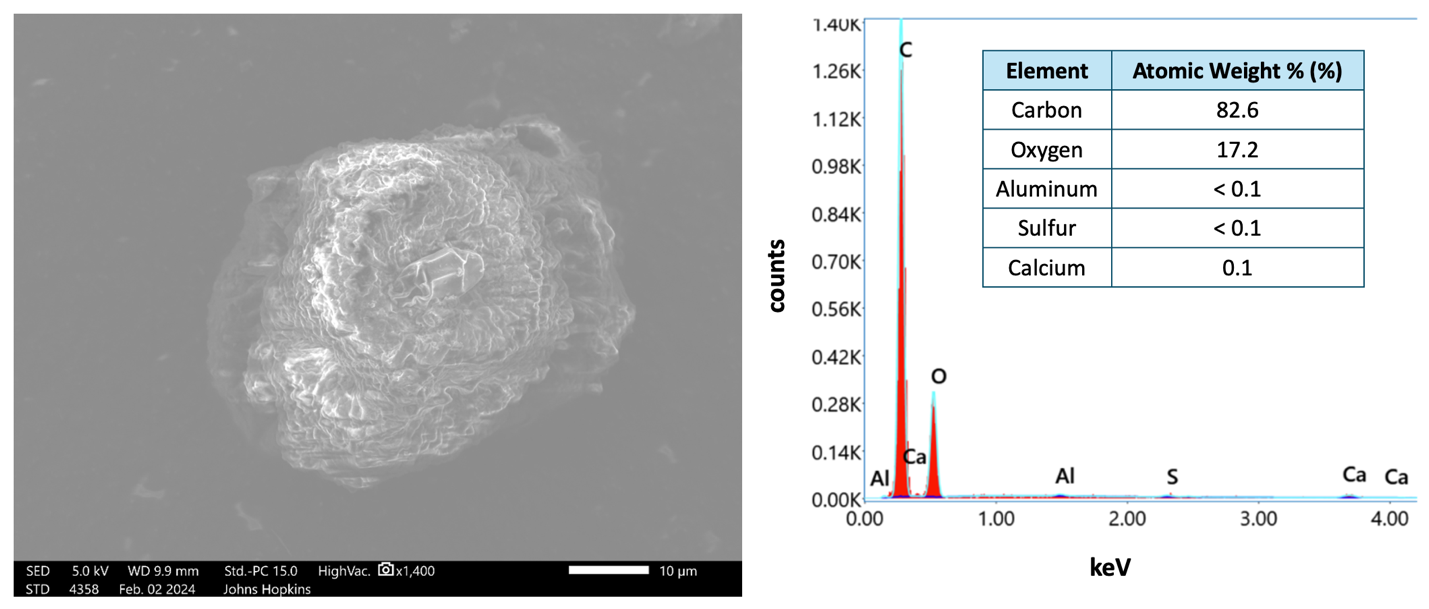


**Text S4. POSITIVELY IDENTIFIED COAL PARTICLES OBSERVED AT LOCATION B**

Location B was the most distal sampling location to the open-air coal terminal in Curtis Bay at 1,235 m from the facility. Six coal particles were postively identified on the sample collected at this location. See main text Figure 4 and Supplementary Figures S15-S19 for all coal particle images and EDX spectra**.**

**Supplementary Figure S11.** High magnification image (secondary electrons) (left) and elemental composition (right) of one out of six positively identified coal particles observed at Location B. This was the 1^st^ particle analyzed in the 3^rd^ area position.


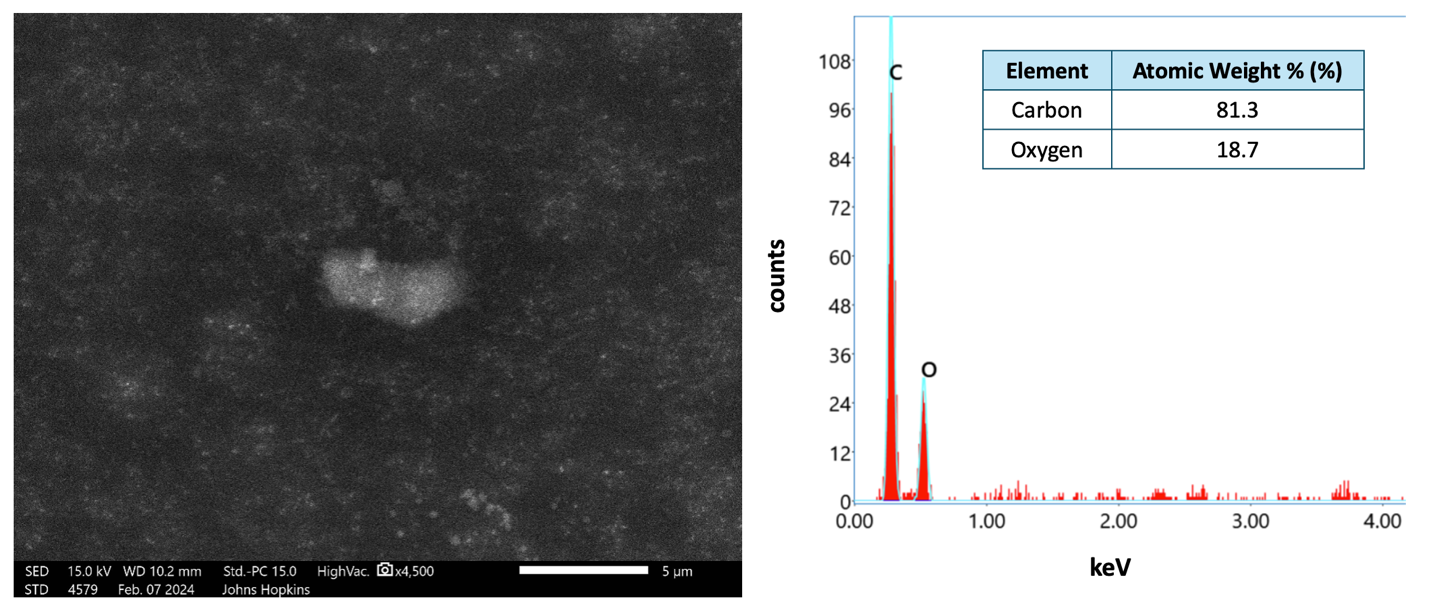


**Supplementary Figure S12.** SEM image (secondary electrons) (left) and elemental composition (right) of one out of six positively identified coal particles observed at Location B (red dot). This was the 10^th^ particle analyzed in the 16^th^ area position. The high magnification image of this particle is unavailable.


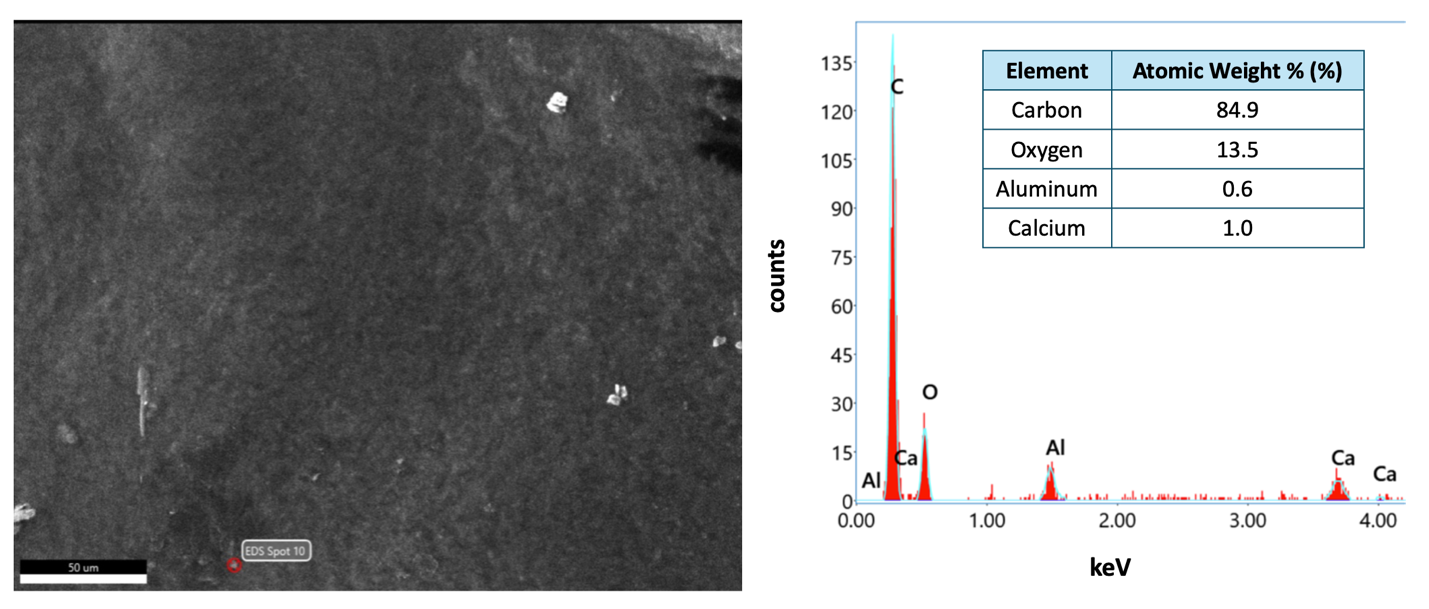


**Supplementary Figure S13.** High magnification image (secondary electrons) (left) and elemental composition (right) of one out of six positively identified coal particles observed at Location B. This was the 1^st^ particle analyzed in the 18^th^ area position.


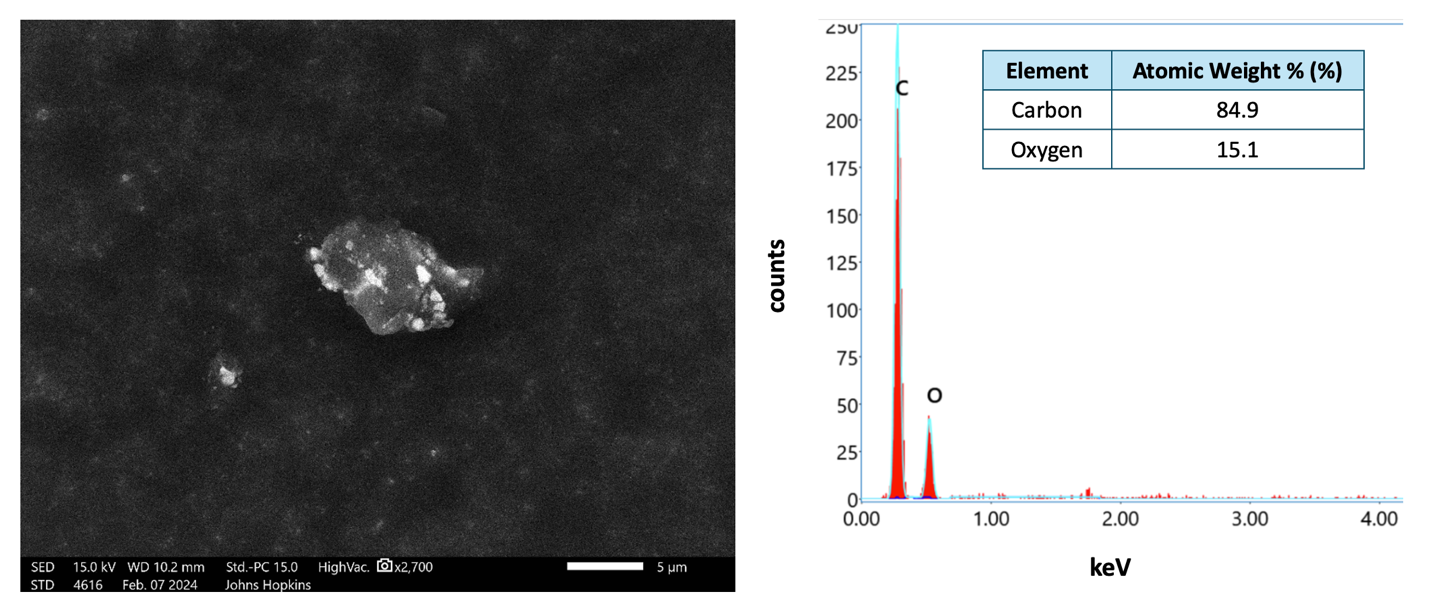


**Supplementary Figure S14.** High magnification image (secondary electrons) (left) and elemental composition (right) of one out of six positively identified coal particles observed at Location B. This was the 5^th^ particle analyzed in the 19^th^ area position.


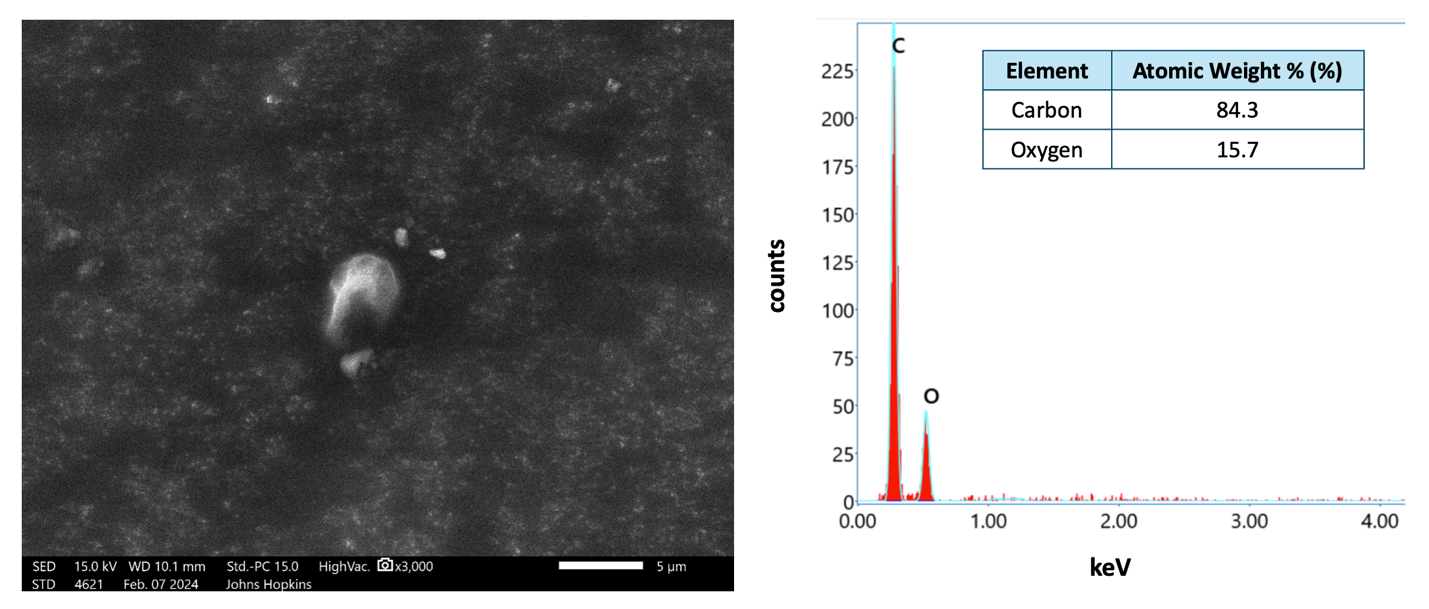


**Supplementary Figure S15.** High magnification image (secondary electrons) (left) and elemental composition (right) of one out of six positively identified coal particles observed at Location B. This was the 6^th^ particle analyzed in the 27^th^ area position.


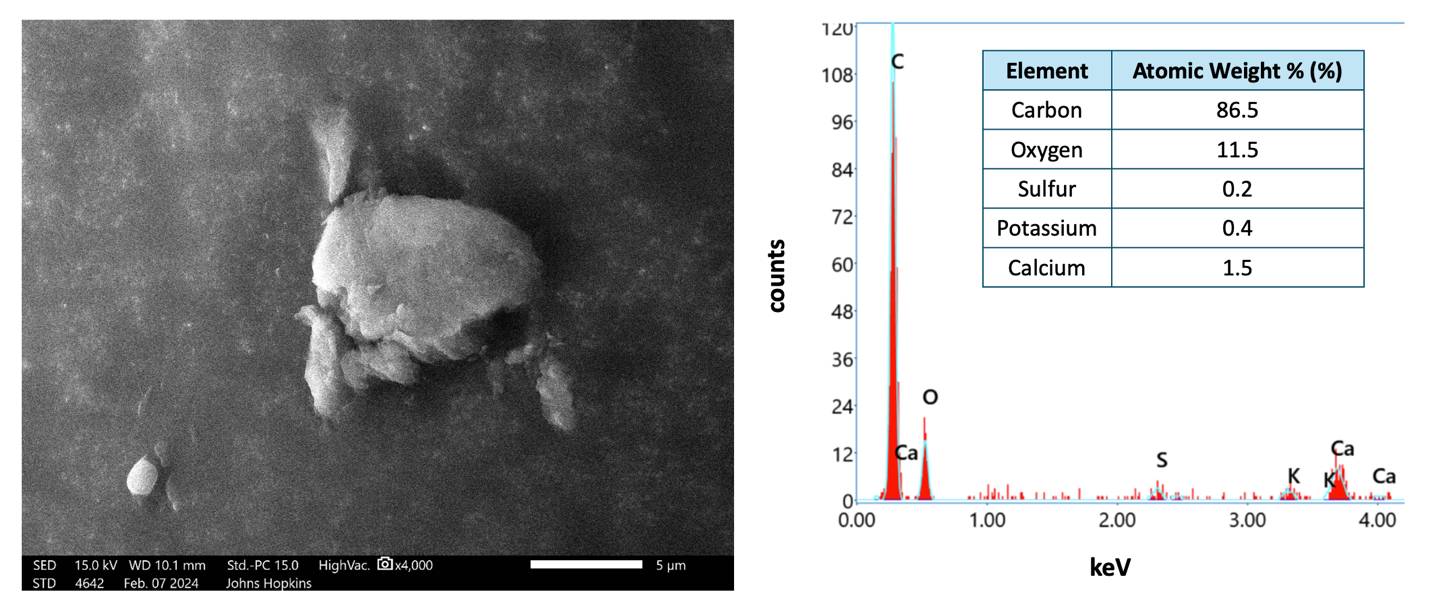

Supplement: Supplementary Material [file NIHMS2029316-supplement-Supplementary_Material.docx]
